# Supplementary material for: Effectiveness of community health workers involvement in smoking cessation programme: A systematic review
Source: PLoS One. 2020 Nov 19;15(11):e0242691. doi: 10.1371/journal.pone.0242691 (PMC7676728; doi:10.1371/journal.pone.0242691)
Supplement: S1 Table — (DOCX) [file pone.0242691.s002.docx]

**S1 Table. Risk of Bias Assessment Details**

| **Study** | **Randomized treatment order (selection bias)** | **Allocation concealment (selection bias)** | **Blinding of participants and personnel (performance bias)** | **Blinding of outcome assessment (detection bias)** | **Incomplete outcome data (attrition bias)** | **Selective outcome reporting (reporting bias)** | **Other bias** |
| --- | --- | --- | --- | --- | --- | --- | --- |
| **Bernstein *et al*, 2011** | On-line random plan generator, allocation ratio 1:1 and a block size of 6 (LOW) | Study personnel (LOW) | Opaque envelope (LOW) | Not mentioned (UNCLEAR) | 17% loss to follow up (LOW) | Protocol unavailable (UNCLEAR) | Measured 7-days self-reported PPA (HIGH) |
| **Wang et al, 2017** | Online generator, block randomization (LOW) | One of the investigators (LOW) | Single blinded (LOW) | Outcome assessors and statistical analysts were blinded (LOW) | 28% loss to follow up (HIGH) | Protocol available, all pre-specified outcome reported (LOW) | Measured validated PPA (LOW) |
| **White et al, 2018** | Qualtrics survey flow randomizer (LOW) | Not mentioned (UNCLEAR) | Not blinded (HIGH) | A blinded outcome assessor (LOW) | 28% loss to follow up (HIGH) | Protocol available, all pre-specified outcome reported (LOW) | Measured 7-days self-reported PPA (HIGH) |
| **Bonevski et al, 2018** | Computer generated randomization (LOW) | Computer programmer (LOW) | Participants were blinded (LOW) | Statistical analysis approved before unblinding (LOW) | 42% loss to follow up (HIGH) | Protocol available, all pre-specified outcome reported (LOW) | Measured verified abstinence (LOW) |
| **Jiang et al, 2018** | Quasi experiment (HIGH) | Quasi Experiment (HIGH) | Not blinded (HIGH) | Not blinded (HIGH) | 12% loss to follow up (LOW) | Protocol unavailable (UNCLEAR) | Measured verified abstinence (LOW) |

Abbreviations: PPA- Point-prevalence Abstinence.
