# Supplementary material for: Effectiveness of community health workers involvement in smoking cessation programme: A systematic review
Source: PLoS One. 2020 Nov 19;15(11):e0242691. doi: 10.1371/journal.pone.0242691 (PMC7676728; doi:10.1371/journal.pone.0242691)
Supplement: Attachment — Submitted filename: PONE-D-20-06267_reviewer (1).pdf [file pone.0242691.s003.pdf]

PLOS ONE

Effectiveness of Community Health Workers in Smoking Cessation Programme: A Systematic Review

--Manuscript Draft--

**Manuscript Number:**	PONE-D-20-06267
**Article Type:**	Research Article
**Full Title:**	Effectiveness of Community Health Workers in Smoking Cessation Programme: A Systematic Review
**Short Title:**	Community health workers involvement in smoking cessation
**Corresponding Author:**	siti hafizah zulkiply, M.D<br>Institute for Medical Research<br>Kuala Lumpur, MALAYSIA
**Keywords:**	Community Health Worker; Smoking Cessation; Sustainable Development Goals; Primary Care
**Abstract:**	Background<br><br>Sustainable Development Goals (SDG) has set the target to reduce by one-third premature mortalities from non-communicable diseases (NCD). One of the ways to achieve this is through strengthening countries' implementation of the World Health Organization Framework Convention on Tobacco Control (WHO FCTC). Community health workers (CHWs) involvement has showed promising results in prevention of NCDs. This systematic review aimed at critically evaluating the available evidences on the effectiveness of involvement of CHWs in smoking cessation.<br><br>Materials and Methods<br><br>We systemically searched PubMed and CENTRAL up to September 2019. We searched for published interventional studies on smoking cessation interventions using usual care that complemented with CHWs as compared to usual or standard care alone. Our primary outcome is abstinence of smoking. Two reviewers independently extracted data and assessed study risks of bias.<br><br>Result<br><br>We identified 2794 articles, with only five included studies. A total of 3512 smokers with 41 CHWs were included in the studies. Intervention duration range from 6 weeks to 30 months. The studies used behavioral intervention or combination of behavioral intervention and pharmacological treatment. Overall, smoking cessation intervention that incorporates involvement of CHWS has higher smoking cessation rates [OR 1.95, 95% CI (1.35,2.82)]. Significant smoking cessation rates were seen in two studies.<br><br>Conclusion<br><br>Higher smoking cessation rates are seen in interventions that combined usual care with interventions by CHWs as compared to usual care alone. Therefore, CHWs have the potential to strengthen WHO FCTC and reduce the NCD mortalities attributed by smoking which fulfills the SDG target 3.4 and 3a.
**Order of Authors:**	siti hafizah zulkiply, M.D
	Lina Farhana Ramli
	Zul Aizat Mohamad Fisal
	Bushra Tabassum
**Additional Information:**	
**Question**	**Response**

Powered by Editorial Manager® and ProduXion Manager® from Aries Systems Corporation

**Financial Disclosure**<br><br>Enter a financial disclosure statement that describes the sources of funding for the work included in this submission. Review the submission guidelines for detailed requirements. View published research articles from *PLOS ONE* for specific examples.<br><br>This statement is required for submission and **will appear in the published article** if the submission is accepted. Please make sure it is accurate.<br><br>**Unfunded studies**<br>Enter: *The author(s) received no specific funding for this work.*<br><br>**Funded studies**<br>Enter a statement with the following details:<br>• Initials of the authors who received each award<br>• Grant numbers awarded to each author<br>• The full name of each funder<br>• URL of each funder website<br>• Did the sponsors or funders play any role in the study design, data collection and analysis, decision to publish, or preparation of the manuscript?<br>• **NO** - Include this sentence at the end of your statement: *The funders had no role in study design, data collection and analysis, decision to publish, or preparation of the manuscript.*<br>• **YES** - Specify the role(s) played.<br><br>* typeset	Authors received no specific funding
**Competing Interests**<br><br>Use the instructions below to enter a competing interest statement for this submission. On behalf of all authors, disclose any competing interests that could be perceived to bias this work—acknowledging all financial support and any other relevant financial or non-financial competing interests.<br><br>This statement **will appear in the published article** if the submission is accepted. Please make sure it is	We declared no competing interests

Powered by Editorial Manager® and ProduXion Manager® from Aries Systems Corporation

accurate. View published research articles from *PLOS ONE* for specific examples.<br><br>**NO authors have competing interests**<br><br>Enter: *The authors have declared that no competing interests exist.*<br><br>**Authors with competing interests**<br><br>Enter competing interest details beginning with this statement:<br><br>*I have read the journal's policy and the authors of this manuscript have the following competing interests: [insert competing interests here]*<br><br>* typeset	
**Ethics Statement**<br><br>Enter an ethics statement for this submission. This statement is required if the study involved:<br><br>• Human participants<br>• Human specimens or tissue<br>• Vertebrate animals or cephalopods<br>• Vertebrate embryos or tissues<br>• Field research<br><br>Write "N/A" if the submission does not require an ethics statement.<br><br>General guidance is provided below. Consult the submission guidelines for detailed instructions. **Make sure that all information entered here is included in the Methods section of the manuscript.**	N/A

**Format for specific study types**<br><br>**Human Subject Research (involving human participants and/or tissue)**<br>• Give the name of the institutional review board or ethics committee that approved the study<br>• Include the approval number and/or a statement indicating approval of this research<br>• Indicate the form of consent obtained (written/oral) or the reason that consent was not obtained (e.g. the data were analyzed anonymously)<br><br>**Animal Research (involving vertebrate animals, embryos or tissues)**<br>• Provide the name of the Institutional Animal Care and Use Committee (IACUC) or other relevant ethics board that reviewed the study protocol, and indicate whether they approved this research or granted a formal waiver of ethical approval<br>• Include an approval number if one was obtained<br>• If the study involved *non-human primates*, add *additional details* about animal welfare and steps taken to ameliorate suffering<br>• If anesthesia, euthanasia, or any kind of animal sacrifice is part of the study, include briefly which substances and/or methods were applied<br><br>**Field Research**<br><br>Include the following details if this study involves the collection of plant, animal, or other materials from a natural setting:<br>• Field permit number<br>• Name of the institution or relevant body that granted permission	
**Data Availability**<br><br>Authors are required to make all data underlying the findings described fully available, without restriction, and from the time of publication. PLOS allows rare exceptions to address legal and ethical concerns. See the PLOS Data Policy and FAQ for detailed information.	Yes - all data are fully available without restriction

A Data Availability Statement describing where the data can be found is required at submission. Your answers to this question constitute the Data Availability Statement and **will be published in the article**, if accepted.<br><br>**Important:** Stating 'data available on request from the author' is not sufficient. If your data are only available upon request, select 'No' for the first question and explain your exceptional situation in the text box.<br><br>Do the authors confirm that all data underlying the findings described in their manuscript are fully available without restriction?	
**Describe where the data may be found in full sentences. If you are copying our sample text, replace any instances of XXX with the appropriate details.**<br><br>• If the data are **held or will be held in a public repository**, include URLs, accession numbers or DOIs. If this information will only be available after acceptance, indicate this by ticking the box below. For example: *All XXX files are available from the XXX database (accession number(s) XXX, XXX.).*<br>• If the data are all contained **within the manuscript and/or Supporting Information files**, enter the following: *All relevant data are within the manuscript and its Supporting Information files.*<br>• If neither of these applies but you are able to provide **details of access elsewhere**, with or without limitations, please do so. For example:<br><br>*Data cannot be shared publicly because of [XXX]. Data are available from the XXX Institutional Data Access / Ethics Committee (contact via XXX) for researchers who meet the criteria for access to confidential data.*<br><br>*The data underlying the results presented in the study are available from (include the name of the third party*	all relevant data are within the manuscript and its supporting information files

and contact information or URL).<br>• This text is appropriate if the data are owned by a third party and authors do not have permission to share the data.<br><br>* typeset	
Additional data availability information:	

Powered by Editorial Manager® and ProduXion Manager® from Aries Systems Corporation

Manuscript Click here to access/download;Manuscript;Manuscript V1.docx

Effectiveness of Community Health Workers Involvement in Smoking Cessation Programme; A Systematic Review.

Siti Hafizah Zulkiply[1*], Lina Farhana Ramli[1], Zul Aizat Mohamad Fisal[1], Bushra Tabassum[1],

[1] Master of Public Health Candidate, Faculty of Medicine and Health Sciences, University Putra Malaysia
*Corresponding author: Siti Hafizah Binti Zulkiply, hafizahzulkiply.hz@gmail.com

ABSTRACT

Background: Sustainable Development Goals (SDG) has set the target to reduce by one-third premature mortalities from non-communicable diseases (NCD). One of the ways to achieve this is through strengthening countries' implementation of the World Health Organization Framework Convention on Tobacco Control (WHO FCTC). Community health workers (CHWs) involvement has showed promising results in prevention of NCDs. This systematic review aimed at critically evaluating the available evidences on the effectiveness of involvement of CHWs in smoking cessation.

Materials and Methods: We systemically searched PubMed and CENTRAL up to September 2019. We searched for published interventional studies on smoking cessation interventions using usual care that complemented with CHWs as compared to usual or standard care alone. Our primary outcome is abstinence of smoking. Two reviewers independently extracted data and assessed study risks of bias.

Result: We identified 2794 articles, with only five included studies. A total of 3512 smokers with 41 CHWs were included in the studies. Intervention duration range from 6 weeks to 30 months. The studies used behavioral intervention or combination of behavioral intervention and pharmacological treatment. Overall, smoking cessation intervention that incorporates involvement of CHWS has higher smoking cessation rates [OR 1.95, 95% CI (1.35,2.82)]. Significant smoking cessation rates were seen in two studies.

Conclusion: Higher smoking cessation rates are seen in interventions that combined usual care with interventions by CHWs as compared to usual care alone. Therefore, CHWs have the potential to strengthen WHO FCTC and reduce the NCD mortalities attributed by smoking which fulfills the SDG target 3.4 and 3a.

Keywords: Community Health Worker, Smoking Cessation, Sustainable Development Goals, Primary Care

Introduction

Non-communicable diseases (NCD) is the major cause of mortalities worldwide. Smoking is an important risk factor for the development of NCD, including cancers, cardiovascular and respiratory diseases. The United Nation General Assembly has developed Agenda for Sustainable Developmental Goals (SDG) in 2015 that contains a total of 17 Goals that all Member States have agreed to achieve by 2030 (1). SDG 3 with goal to 'ensure healthy lives and promoting well-being for all ages', includes target 3.4 which is to reduce by one-third premature mortality from NCD and target 3a which is to strengthen country implementation of the World Health Organization Framework Convention on Tobacco Control (WHO FCTC) (2).

In September 2005, Malaysia had parted with WHO FCTC and agreed to fulfil the demand to develop and disseminate a standard national guideline on tobacco (3). In response, Malaysian government had set up a target to reduce 30% relative reduction of national smoking prevalence by 2025 within the National Strategic Plan for NCD (NSP-NCD). In addition, World Health Organization (WHO) has also developed MPOWER that include measures for demand reduction which were; i) Monitoring tobacco use and prevention policies, ii) Protecting people from tobacco smoke, iii) Offer help to quit tobacco use, iv) Warn about the dangers of tobacco, v) Enforce bans on tobacco advertising, promotion and sponsorship and vi) Raise taxes on tobacco (4).

Tobacco has become a major public health enemy, accountable for more than 7 million preventable deaths yearly, and forecasted that more than 8 million people will die from diseases related to tobacco use by year 2030 if pattern of smoking continues (5). In Malaysia alone, it is estimated that more than 20,000 died due to tobacco each year (6). There are 933.1 million smokers worldwide in 2015 and around 80% of them live in Low and Middle-income

Countries (LMICs) (World Health Organization Media Center, 2017). According to National Health and Morbidity Survey (NHMS) 2015, the prevalence of smokers in Malaysia were 22.8%, therefore, it is estimated that nearly five million Malaysians aged 15 years and above are smokers (3). The mean age of smoking initiation was 18.3 years (8), with the highest prevalence of smokers were among 25 to 44 years old age group (28%), followed by 45 to 64 years old age group (20%) (3).

In many countries, smoking cessation programme (identification of smokers, advised and offered quit support) are taking place in primary care setting (9). Evidence for the effectiveness of these intervention in this setting given by professional healthcare is well established (10). Malaysia has set up quit smoking clinic since 2004 that is available at almost all primary health clinics (11). Unfortunately, studies have also shown that high mortalities in low socioeconomic area, especially secondary to NCD are due to barriers in healthcare services (12). In view of low socioeconomic status being an important determinant of smoking (13), therefore, it is pertinent that focus should be given on interventions in smoking cessation in these area. As reported by NHMS 2015, the prevalence of smokers in rural areas were higher (27.7%) as compared to urban area (20.9%) (3). WHO has developed an effective community-based strategy to reduce the gap in healthcare in low-socioeconomic areas. Furthermore, current smoking cessation services were not felt to be user-friendly and were poorly understood (14).

In line with the Alma Ata declaration of Primary Healthcare Concept, all community health strategy designs must address community needs at the local level and be led by the community members themselves, and require involvement of communities to mobilize local resources. Task shifting, an idea conceived by the WHO, defined as redistributing primary

care responsibilities from physician to non-physician providers (15). Task shifting can be further extended to include health workers without formal healthcare training, known as community health workers (CHWs) (16). In 1989 a WHO study group had established a widely accepted definition of CHWs that mentioned CHWs should be among the communities where they practice, should be chosen by the communities, should be able to answer to the public for their programs, should be supported by the health system, and have less duration of training than professional workers (17). CHWs have the potential to achieve primary health care goals, by enhancing access to care and promote a proper use of health resources through the provision of cultural and outreach links between communities and health systems, giving health education to reduce cost, screening services, basic emergency services, continuum of care and client protection (17). Furthermore, it is pertinent to enhance civic engagement and ensure accountability in order to achieve SDG goals (18,19). Numerous studies have evaluated the role of community intervention on major cardiovascular events and risk factors. Community based cardiovascular health intervention in vulnerable populations showed that interventions aimed at decreasing blood pressure were the most promising and behavior change interventions were the most challenging (20,21).

Guidelines on smoking cessation programme showed that combination of behavioral change and pharmacological support given by professional healthcare are the most effective. CHWs is crucial as they serve as critical link in increasing communities' access to services, especially for people living in rural and undeserved areas, and ultimately an integral part needed to achieve SDG goals. Therefore, the aim of this study is to evaluate the effectiveness of involvement of CHWs in smoking cessation as compared to usual care.

Materials and Methods

This review was conducted and reported in accordance to the Preferred Reporting Items for Systematic Reviews and Meta-Analyses (PRISMA) guidelines and based on the Cochrane Collaboration approach.

2.1 Eligibility criteria

We included intervention studies that compared combination of interventions given by CHWs and usual care with only usual care. We included studies that were conducted among smokers aged 18 years old and above. We included studies with abstinence of smoking as the outcome. We excluded studies that conducted intervention in adolescent or special group (define as mental illness or LGBTQ). Non-English language studies, reviews, proceedings, qualitative studies, descriptive studies and protocol were also excluded.

2.2 Data Sources and search strategy

We systematically searched for relevant articles published in The Cochrane Central Register of Controlled Trials (CENTRAL) and PubMed/MEDLINE up to September 2019. Search were limited to 10 years. We combine the keywords for smoking, cessation and community as the following: cessation or reduction AND smoking or tobacco AND volunteer or peer or community or lay.

2.3 Study selection

A pair of authors independently assessed the titles and abstracts of a defined set of articles. Each study was recorded as include, exclude or unclear. Full articles were retrieved for further assessment if they were recorded as include or unclear. Eligible studies were identified based on the inclusion criteria. Any discrepancies in the assessment were resolved by discussion leading to a consensus.

2.4 Data Extraction, Data Analysis and Risk of Bias assessment

A table for data extraction from all potential studies were built. The table included information on study characteristics (sample size and study duration), participant characteristics (setting and population type), intervention characteristics (type of interventions given in both arms), training of CHWs (background, duration of training and training module) and analysis and results (outcomes). All authors independently extracted the data and any discrepancies were resolved by discussion. The characteristics of the included studies are outlined in Table 1, Table 2, Table 3 and Table 4.

Data synthesis and analysis were carried out using Review Manager Software (Rev Man) version 5.3 (Nordic Cochrane Centre, Cochrane Collaboration, Copenhagen). End of intervention values were taken. If studies measured smoking cessation rates at multiple intervals (3 or 6 or 9 months), outcomes on the final point of interval was taken. If studies reported two types of smoking cessation measurements (self-reported or validated), validated outcomes were taken.

We assessed the study quality of each included study using the COCHRANE guideline for assessment of systematic reviews and the published guide on risk of bias assessment for intervention studies. The studies were evaluated based on eight criteria: randomized treatment order, allocation concealment, blinding of participants and personnel, blinding of outcome assessment, incomplete outcome data, selective outcome reporting and other bias. For each item, risk of bias was classified as ‘low risk’, ‘high risk, or ‘unclear risk’, with the last category indicating either lack of information or uncertainty over the potential for bias. Results were presented in a ’Risk of bias’ summary (Figure 3a and 3b).

Result

3.1 Search results

We identified 2794 articles through our electronic databases search. A total of 2694 articles were excluded *following* titles and abstracts screening due to irrelevant study designs, irrelevant study populations, outcomes, and intervention characteristics. 100 full text articles were screened, of which 95 articles were excluded for following reasons; interventions were not given by CHWs (73), study designs were non-randomized controlled trials (17) and outcomes were not smoking cessation (5). Finally, five studies are included in this review (22–26). PRISMA Flowchart can be found at Figure 1.

Figure 1: PRISMA flowchart for the selection of studies. Outcomes of the systematic review of the literature by record identification, screening, and analysis in the Preferred Reporting Items for Systematic Reviews and Meta-Analyses (PRISMA) statement flow diagram.

3.2 Characteristics of included studies

A total of 3512 participants are included in this review. Studies were conducted between the year of 2011 and 2018. Two studies were conducted in United States of America (USA) (22,23), while other studies were conducted in Hong Kong (24), Australia (26) and Vietnam (25) respectively. Duration of the studies conduct were ranging from 6 weeks to 30 months. All studies were conducted among community except for one study that conducted in emergency department setting (22). One study were targeted on specific ethnicity (African American and Hispanic community) (22). There were no significant differences between number of cigarettes per day (13.08 ± 9.57).

Table 1. Characteristic of studies

Study	Setting	Participants type	Sample size	Intervention	Control	Mean cigarettes/ day	Study Design	Study Duration
**Bernstein *et al*, 2011** (22)	Bronx, New York, United States of America	Hispanic and African American	337	170	167	15 ± 7.48	RCT	21 months
**Wang *et al*, 2017** (24)	Hong Kong	Public	1,226	402	416<br>408	N/A	Cluster-3 arms RCT	3 months
**White *et al*, 2018** (23)	Maryland, United States of America	Public	200	101	99	19.3 ± 18.3	RCT	6 – 8 weeks
**Bonevski *et al*, 2018** (26)	New South Wales, Australia	Disadvantage Adult clients of the Community Care Centre	431	187	244	15 ± 7.46	Parallel randomised trial	30 months
**Jiang *et al*, 2018** (25)	Thai Nguyen, Vietnam	Village Population	1,318	781	537	11.02 ± 9.48	RCT and Quasi-experimental	6 months

Abbreviations: RCT- randomized controlled trial

3.3 Characteristic of Intervention

All of the studies used behavioral intervention as the method for smoking cessation. The usual care intervention that were given to both intervention groups and control group are mainly brief advice, and some of the studies provided self-help material. Four studies used brief advice; one study used AWARD model (Ask, Warn, Advise, Refer and Do-it-again) (24), one study used 4A's model (Ask screen for tobacco use, Advise to quit, Assess readiness to quit, and Assist) (25), one study used automated Smokefree TXT (23) and finally one study used on screen advice (26). Self-help material in the form of leaflet or brochure was provided to the participants in three studies (22,24,25).

The interventions given by CHWs by the studies were behavioral intervention, active referral and pharmacological therapy. Two studies used motivational interviewing (MI) and provided pharmacological treatment (nicotine replacement therapy (NRT)) to the participants (22,26). One study had active referral to existing smoking cessation intervention in clinics by CHWs (24). One study used personalized text messages (23) as mediator for smoking cessation intervention. Details of the study interventions can be found in Table 2.

3.4 Smoking Abstinence Rate

All studies used intention to treat analysis, except for study by Bonevski et al (26). Outcomes of smoking abstinence rate were measured by either self-reported or chemically verified (CO). Two studies provided abstinence rate at 3 months (22,23) and the remaining three studies at 6 months (24–26).

Overall, all studies had higher odds of abstinence in intervention group as compared to usual group. Two studies reported significant smoking abstinence rate in interventions using CHWs with OR 2.96 [2.15, 4.07] ($p<0.001$) (25)and OR 1.81 [1.04, 3.16] (active referral vs brief

advice group), OR 1.85 [1.06, 3.23] (active referral vs control group) (24). Details of the result can be found in Table 2.

Figure 2: Forest plot shows smoking abstinence rate.

Table 2. Characteristic of Intervention and Outcome

Study	Intervention	Control	Outcome Intervention	Outcome Control
**Bernstein *et al*, 2011** (22)	Smoking cessation brochure (10-15 min) AND MI by interventionist, 6 weeks course of NRT.	Smoking cessation brochure and contact information to smoking cessation programs	Self-reported 7-days PPA rates at 3 months: 14.7%<br>OR 1.14 [0.61, 2.11]	Self-reported 7-days PPA rates at three months: 13.2%
**Wang *et al*, 2017** (24)	Brief advice using structural model AWARD, health warning leaflet AND active referral to SC services by ambassadors	Brief advice using structural model AWARD, health-warning leaflet and encourage to SC services	Validated abstinence rates at 6 months: 9.0% (p<0.05)<br>OR 1.81 [1.04, 3.16]<br>OR 1.85 [1.06, 3.23]	Validated abstinence rates at 6 months:<br>5.0% and<br>5.1%
**White *et al*, 2018** (23)	Automated SFTXT<br>AND personalized text messages from peer mentor	Automated SFTXT messages	Self-reported 7-day PPA rates at 3 months: 7.9%<br>OR 2.75 [0.71, 10.70]	Self-reported 7-day PPA rates at 3 months: 3.0%
**Bonevski *et al*, 2018** (26)	On screen advice to quit smoking, state Quitline telephone number, and a gift bag with call it quits<br>AND MI and NRT by trainer volunteer case workers	On screen advice to quit smoking, state Quitline telephone number, and a gift bag with call it quits	Self-reported continuous verified PPA at 6 months: 1.0%<br>OR 0.76 [0.07, 8.44]	Self-reported continuous verified PPA at 6 months: 1.4%
**Jiang *et al*, 2018** (25)	4As (brief counselling and educational materials) AND refer smokers to a trained VHW (4As+R)	4As (brief counselling and educational materials)	Validated abstinence rate at 6 months: 25.7% (p<0.001)<br>OR 2.96 [2.15, 4.07]	Validated abstinence rate at 6 months: 10.5%

Abbreviations: MI- Motivational Interviewing; NRT- nicotine replacement therapy; PPA- point prevalence abstinence; SC- smoking cessation; CHW-Community Health Worker; AWARD; SFTXT- SmokefreeTXT; SC- smoking cessation; 4As- Ask (screen for tobacco use), Advise to quit, Assess readiness to quit, and Assist; VHW- Village Health Worker.

3.5 Characteristics and Training of Community Health Workers (CHWs)

The number of CHWs in the study varied from one person to 32 persons, with two studies did not provide amount of CHWs (24,26). Two studies had C[illegible]Ws from the communities (23,25), one study had university students (24) and one study had volunteers from NGO (26). CHWs from two studies had experienced working as CHWs or working on smoking cessation programs previously (23,25). One study had CHWs whom was former smokers and provided incentives to the CHWs (23). The duration of training of the CHWs ranging from just two hours to two weeks. Training were given by either the researchers or experts in the field. Content of the training were comprised of the study protocol and method in smoking cessation intervention. Supervisions were given weekly or biweekly to ensure fidelity. Details of the training can be found in Table 3.

Table 3: Charateristic of CHWs and training of CHWs.

Table 3. Characteristic of Community Health Worker and Training of Community Health Worker

Study	Amount and Description	Background	Experience	Incentive	Duration/ Training Module/ Supervision
**Bernstein *et al*, 2011** (22)	One (Peer educator)	ED based and former smoker.	Not reported	None	Duration: 2 weeks<br><br>Training module: Course and practicum (roleplay, slide shows and observation)- Epidemiology, health effects and treatment of tobacco dependence, motivational interviewing.<br><br>Supervision: Every two weeks
**Wang *et al*, 2017** (24)	Not mentioned (SC ambassador)	University students (health-related studies) and volunteers from NGO.	Not reported	None	Duration: 4 hours<br><br>Training module: Tobacco control and SC, SC reduction advice skills<br><br>Supervision: Spot checks
**White et al, 2018** (23)	Thirty-two (Peer mentor)	Former smoker more than 1 year, 18 years old and above and lived in the United States, willingness to mentor smokers through texting and completing the online training program.	Facilitators of the American Cancer Society Freshstart group-based cessation support program.	$200 and entry into $1000 drawing	Duration: 2 hours<br><br>Training module: Online- study details, smoking and SC, MI, web-based-text-messaging platform<br><br>Supervision: N/A
**Bonevski et al, 2018** (26)	Not mentioned (Caseworker)	Volunteer case workers of community social service organizations (NGO) in NSW.	Not reported	None	Duration: One day<br><br>Training module: Behavioral counselling and MI<br><br>Supervision: N/A
**Jiang et al, 2018** (25)	Six to eight (VHW)	Not a current smoker and willing to participate in the	Worked as a VHW at that particular site for a year or more.	None	Duration: 4 days<br><br>Training module: Research ethics, study protocol,

Study	Amount and Description	Background	Experience	Incentive	Duration/ Training Module/ Supervision
		required components of the study intervention.			use of CO monitor, theory of behavior change, MI, social cognitive skills building approach.<br><br>Supervision: N/A

Abbreviations: N/A- not available; SC-Smoking Cessation; MI-motivational Interviewing; ED- Emergency Department; NGO-non-governmental organizations; NSW-New South Wales; VHW-village health worker.

3.6 Risk of bias assessment

We judged the risk of possible bias present in the included studies according to the four criteria incorporated. We presented the summary of risk of bias assessment of the included studies in Table 4 and Figure 3(a) and 3(b). Random sequence generation was assessed to be of low risk of bias in all studies except for study by Jiang et al (25) as they did not clearly state the method of random sequence generation. When assessing the allocation concealment of the included studies, three studies had low risk of bias while the other two studies (23,25) did not clearly state the method of allocation of treatment groups. Performance bias was assessed to be low risk of bias in only two studies study (22,26) with the remaining studies assessed to be high risk of bias. Detection bias was assessed to be low risk of bias in two studies (24,26). Only study by Jiang et al.,2018 had low risk in attrition bias (25), the remaining studies were assessed to be high risk of bias due to high dropout rate (>20%). Three studies with available protocol and all pre-specified outcomes measured were considered as low risk of reporting bias (23,24,26).

Figure 3 (a) and 3 (b): Risk of bias assessment.

Discussion

There are considerable evidences supporting impact of CHWs on the health of diverse populations especially on maternal and child health, malaria and tuberculosis. However, evidence of effectiveness of CHWs in cardiovascular diseases (CVD) management and prevention are still lacking. Previous studies reported evidences on the four main risk of CVD; namely hypertension, diabetes mellitus, unhealthy diet and alcohol and tobacco consumption. Task sharing intervention were proven effective in lowering low-density lipoprotein cholesterol (LDL) and total cholesterol (27). In another review, significant reduction in mean blood pressure and glycated haemoglobin levels were seen in task shifting interventions for CVD risk reduction in LMICs (27,28). A study by Jeet et al., 2017 also reported increase in tobacco cessation in interventions using CHWs as compared with standard care (29). Our review has also found similar finding, promising evidence in interventions given by CHWs where higher smoking cessation rates were seen.

WHO has formulated a set of recommendations that provide guidance for the task shifting approaches. These recommendations have implications for a range of health services including management of NCDs. Main enablers of CHWs interventions are provision of algorithms and protocols, while, restrictions on prescribing medications and availability of medicines were main barriers identified.

There are several limitations in this review, first is the high heterogeneity in baseline characteristics of participants and study designs. Theoretical health belief models explained that a person who is in contemplation stage may be easily influence to receive intervention. Therefore, studies with participants with low exposure to smoking and intention to quit participants (theoretical health belief models) might be attributable to higher cessation rates. One study that selected participants who already have the intention to quit smoking showed

similar results (23). Similarly, one study that included participants with low-amount of cigarettes used (one in the past 3 months or 1 months) showed significant smoking cessation rates (24). While, one study that was conducted in hospital setting (emergency department) reported higher abstinence rate in smokers who have smoking-related health issues (22). Review of 33 studies reported high-certainty evidence that incentives improve smoking cessation rates (30). Our review showed similar result, whereby one study that provided incentives to the participants had significant smoking cessation rates (24).

There was also high heterogeneity in the interventions given. Guidelines on smoking cessation programme showed that combination of behavioral change and pharmacological support are the most effective. All of the studies used behavioural intervention as the method for smoking cessation with two studies provided pharmacological treatment (nicotine replacement therapy) to the participants. Our review showed that significant higher abstinence rates were seen in one study that used active referral, AWARD and leaflet (24) and one study that used 4A's (25).

The effectiveness of using CHWs vary depending on their training program, demographics and settings (31). CHWs productivity is determined in large part by the condition under which they work. Attention to the provision of an enabling work environment for CHWs is essential for achieving high levels of productivity. Jaskiewicz et al. present a model in which the work environment encompasses four essential elements, namely workload, supportive supervision, supplies and equipment, and respect from the community and the health system (32). Another important determinant of positive CHWs interventions is community embeddedness whereby community members have a sense of ownership of the program (33). Significant smoking cessation rates were seen in two studies that conducted the training in 4 hours (24) and 4 days

(25) respectively. However, other factors have to be considered. Performance of CHWs were also associated with their age, skill and educational level (34). Study that reported significant higher cessation rate had university students as the CHWs (Wang et al., 2017). While, another study that reported significant higher cessation rate had CHWs from the community and volunteer from NGOs(25). The CHWs from this study had experienced working as CHWs previously (25) .

Higher retention was significantly associated with higher quit rates. However, there were high attrition rates (loss of follow up >20%) in most of the studies included in this review, with the highest attrition rates of 42% (26). Reviews reported that increasing age, higher level of education and higher motivation to quit were associated with higher retention (37).

Recommendations

Previous review has reported cost-effectiveness of CHW interventions as compared to standard care particularly in tuberculosis, malaria and maternal and child health (38,39). We recommend that future studies analyze the cost-effectiveness of using CHWs in smoking cessation. In view of the effectiveness of CHWs intervention is largely dependent on framework and training module, therefore, it is pivotal that guideline on the framework is drawn up. We also recommend that studies measures abstinence rates of smoking according to gold standard.

Conclusion

CHWs have the potential to be a bridge between primary healthcare and communities, and therefore reduce the gap especially among low socioeconomic populations as recommend by

SDG. Our review reported positive outcome on using combination of CHWs and usual care in smoking cessation as compared with usual care alone.

Acknowledgement

We would like to thank the Director General of Health Malaysia, Director of Institute for Medical Research for permission to publish this article. We would also would like to thank Prof. Madya Dr. Rosliza Abdul Manaf for the guidance and support.

Declaration

The authors declare no conflict of interest.

Funding

The authors received no specific funding.

Supplementary Information

S1 PRISMA Checklist

S2 Risk of Bias Details

Authors contribution

Siti Hafizah Zulkiply: Analysis, Writing- Original draft and final editing.

Lina Farhana Ramli: Analysis- Writing - Original draft

Zul Aizat Mohamad Fisal: Analysis, Writing- Original draft

Bushra Tabassum: Analysis, Writing- Original Draft

References

1. United Nations. Transforming our world: the 2030 Agenda for Sustainable Development. United Nations Sustainable knowledge platform. Sustain Dev Goals. 2015;
2. World Health Organisation. Who Framework Convention. WHO Framew Conv Tob Control [Internet]. 2003;42. Available from: http://www.who.int/tobacco/framework/WHO_FCTC_english.pdf
3. Institute for Public Health. National Health and Morbidity Survey 2015 (NHMS 2015). Ministry of Health Malaysia. 2015.
4. World Health Organization. MPOWER : Six policies to reverse the tobacco epidemic. WHO Rep Glob Tob Epidemic. 2008;23–41.
5. WHO. Monitoring tobacco use and prevention policies fresh and alive. 2017. 1–135 p.
6. Ministry of Health Malaysia. Clinical Practice Guidelines on Treatment of Tobacco Use Disorder. Vol. 91, 感染症誌. 2016. 102 p.
7. Wolrd Health Organization Media Center. WHO Fact Sheet | Tobacco. Wolrd Health organization Fact Sheet. 2017.
8. Lim HK, Ghazali SM, Kee CC, Lim KK, Chan YY, Teh HC, et al. Epidemiology of smoking among Malaysian adult males: Prevalence and associated factors. BMC Public Health. 2013;13(1):2–11.
9. Piné-Abata H, Mcneill A, Murray R, Bitton A, Rigotti N, Raw M. A survey of tobacco dependence treatment services in 121 countries. Addiction. 2013;108(8):1476–84.
10. Stead LF, Buitrago D, Preciado N, Sanchez G, Hartmann-Boyce J, Lancaster T. Physician advice for smoking cessation. Cochrane Database of Systematic Reviews. 2013.
11. Fai SC, Yen GK, Malik N. Quit rates at 6 months in a pharmacist-led smoking cessation service in Malaysia. Can Pharm J. 2016 Sep;149(5):303–12.
12. Yusuf S. Improving worldwide access to inexpensive and effective treatments for common cardiovascular diseases. Eur Hear Journal, Suppl. 2018;20:C18–22.
13. Hiscock R, Dobbie F, Bauld L. Smoking cessation and socioeconomic status: An update of existing evidence from a national evaluation of english stop smoking services. Biomed Res Int. 2015;2015.
14. Chean KY, Goh LG, Liew KW, Tan CC, Choi XL, Tan KC, et al. Barriers to smoking cessation: A qualitative study from the perspective of primary care in Malaysia. BMJ Open. 2019;9(7):1–8.
15. World Health Organization. Task Shifting- Global Recommendations & Guidelines. World Heal Organ. 2008;
16. Fulton BD, Scheffler RM, Sparkes SP, Auh EY. Human Resources for Health Health workforce skill mix and task shifting in low. Hum Resour Health. 2011;1–11.
17. Lehmann U, Sanders D. Community health workers: What do we know about them? 2007.
18. Buse K, Hawkes S. Health in the sustainable development goals: Ready for a paradigm shift? Global Health. 2015;11(1):1–8.
19. Joshi R, Peiris D. Task-sharing for the prevention and control of non-communicable diseases. Lancet Glob Heal [Internet]. 2019;7(6):e686–7. Available from: http://dx.doi.org/10.1016/S2214-109X(19)30161-5
20. Joshi R, Thrift AG, Smith C, Praveen D, Vedanthan R, Gyamfi J, et al. Task-shifting for cardiovascular risk factor management: Lessons from the Global Alliance for Chronic Diseases. BMJ Glob Heal. 2018;3:1–8.
21. Anand TN, Joseph LM, Geetha A V., Prabhakaran D, Jeemon P. Task sharing with non-physician health-care workers for management of blood pressure in low-income and middle-income countries: a systematic review and meta-analysis. Lancet Glob Heal [Internet]. 2019;7(6):e761–71. Available from: http://dx.doi.org/10.1016/S2214-109X(19)30077-4
22. Bernstein SL, Bijur P, Cooperman N, Jearld S, Arnsten JH, Moadel A, et al. A randomized trial of a multicomponent cessation strategy for emergency department smokers. Acad Emerg Med. 2011;
23. White JS, Toussaert S, Thrul J, Bontemps-Jones J, Abroms L, Westmaas JL. Peer Mentoring

and Automated Text Messages for Smoking Cessation: A Randomized Pilot Trial. Nicotine Tob Res. 2019;1–10.
24. Wang MP, Suen YN, Li WHC, Lam COB, Wu SY Da, Kwong ACS, et al. Intervention with brief cessation advice plus active referral for proactively recruited community smokers: A pragmatic cluster randomized clinical trial. JAMA Intern Med. 2017;177(12):1790–7.
25. Jiang N, Siman N, Cleland CM, Van Devanter N, Nguyen T, Nguyen N, et al. Effectiveness of Village Health Worker–Delivered Smoking Cessation Counseling in Vietnam. Nicotine Tob Res. 2018;1–7.
26. Bonevski B, Twyman L, Paul C, D'Este C, West R, Siahpush M, et al. Smoking cessation intervention delivered by social service organisations for a diverse population of Australian disadvantaged smokers: A pragmatic randomised controlled trial. Prev Med (Baltim) [Internet]. 2018;112(November 2017):38–44. Available from: https://doi.org/10.1016/j.ypmed.2018.04.005
27. Anand TN, Joseph LM, Geetha A V., Chowdhury J, Prabhakaran D, Jeemon P. Task-sharing interventions for cardiovascular risk reduction and lipid outcomes in low- and middle-income countries: A systematic review and meta-analysis. J Clin Lipidol. 2018;12(3):626–42.
28. Ogedegbe G, Gyamfi J, Plange-Rhule J, Surkis A, Rosenthal DM, Airhihenbuwa C, et al. Task shifting interventions for cardiovascular risk reduction in low-income and middle-income countries: A systematic review of randomised controlled trials. BMJ Open. 2014;4(10).
29. Jeet G, Thakur JS, Prinja S, Singh M. Community health workers for noncommunicable diseases prevention and control in developing countries: Evidence and implications. PLoS One [Internet]. 2017;12(7):1–21. Available from: http://dx.doi.org/10.1371/journal.pone.0180640
30. Notley C, Gentry S, Livingstone-Banks J, Bauld L, Perera R, Hartmann-Boyce J. Incentives for smoking cessation. Cochrane Database of Systematic Reviews. 2019.
31. Abdel-All M, Putica B, Praveen D, Abimbola S, Joshi R. Effectiveness of community health worker training programmes for cardiovascular disease management in low-income and middle-income countries: A systematic review. BMJ Open. 2017;7(11):1–11.
32. Jaskiewicz W, Tulenko K. Increasing community health worker productivity and effectiveness: A review of the influence of the work environment. Hum Resour Health. 2012;10:1–9.
33. Scott K, Beckham SW, Gross M, Pariyo G, Rao KD, Cometto G, et al. What do we know about community-based health worker programs? A systematic review of existing reviews on community health workers. Hum Resour Health. 2018;16(1):1–17.
34. Chung MHL, Hazmi H, Cheah WL. Role performance of community health volunteers and its associated factors in Kuching district, Sarawak. J Environ Public Health. 2017;2017.
35. Louwagie GMC, Okuyemi KS, Ayo-Yusuf OA. Efficacy of brief motivational interviewing on smoking cessation at tuberculosis clinics in Tshwane, South Africa: A randomized controlled trial. Addiction. 2014;109(11):1942–52.
36. Begh RA, Aveyard P, Upton P, Bhopal RS, White M, Amos A, et al. Promoting smoking cessation in Pakistani and Bangladeshi men in the UK: Pilot cluster randomised controlled trial of trained community outreach workers. Trials [Internet]. 2011;12(1):197. Available from: http://www.trialsjournal.com/content/12/1/197
37. Courtney RJ, Clare P, Boland V, Martire KA, Bonevski B, Hall W, et al. Predictors of retention in a randomised trial of smoking cessation in low-socioeconomic status Australian smokers. Addict Behav [Internet]. 2017;64:13–20. Available from: http://dx.doi.org/10.1016/j.addbeh.2016.07.019
38. Nkonki L, Tugendhaft A, Hofman K. A systematic review of economic evaluations of CHW interventions aimed at improving child health outcomes. Hum Resour Health. 2017;15(1):1–19.
39. Vaughan K, Kok MC, Witter S, Dieleman M. Costs and cost-effectiveness of community health workers: Evidence from a literature review. Hum Resour Health. 2015;13(1).

Figure

Click here to access/download;Figure;Figure 1.tif

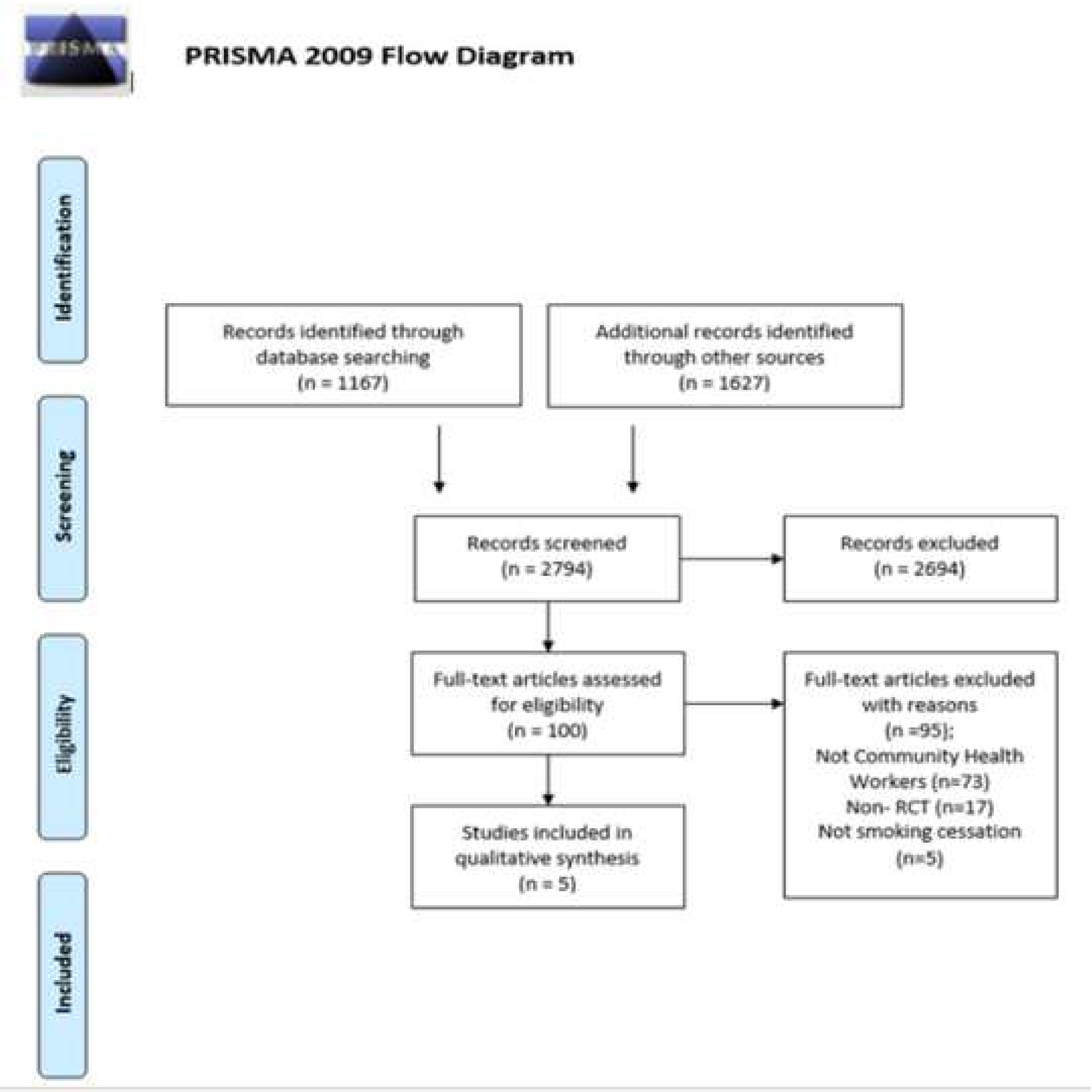

Figure

Click here to access/download;Figure;Figure 2.tif

	CHW		Control			Odds Ratio
Study or Subgroup	Events	Total	Events	Total	Weight	M-H, Random, 95% CI
Bernstein et al, 2011	25	170	22	167	18.8%	1.14 [0.61, 2.11]
Bonevski et al, 2018	1	99	2	150	2.2%	0.76 [0.07, 8.44]
Jiang et al, 2018	200	781	56	537	30.9%	2.96 [2.15, 4.07]
Wang et al, 2017	36	402	21	416	20.9%	1.85 [1.06, 3.23]
Wang et al, 2017	36	402	21	408	20.9%	1.81 [1.04, 3.16]
White et al, 2018	8	101	3	99	6.2%	2.75 [0.71, 10.70]
**Total (95% CI)**		**1955**		**1777**	**100.0%**	**1.95 [1.35, 2.82]**
Total events	306		125			

Heterogeneity: $Tau^2 = 0.09$; $Chi^2 = 9.39$, df = 5 (P = 0.09); $I^2 = 47\%$

Test for overall effect: Z = 3.58 (P = 0.0003)

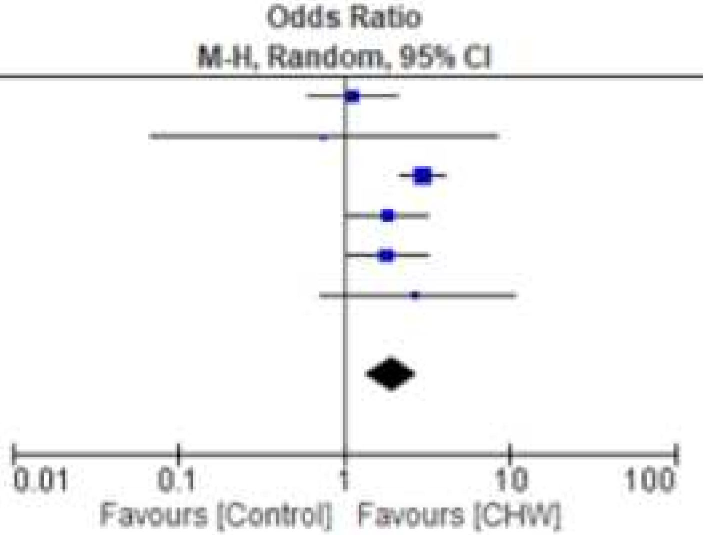

Figure

Click here to access/download;Figure;Figure 3(a).tif

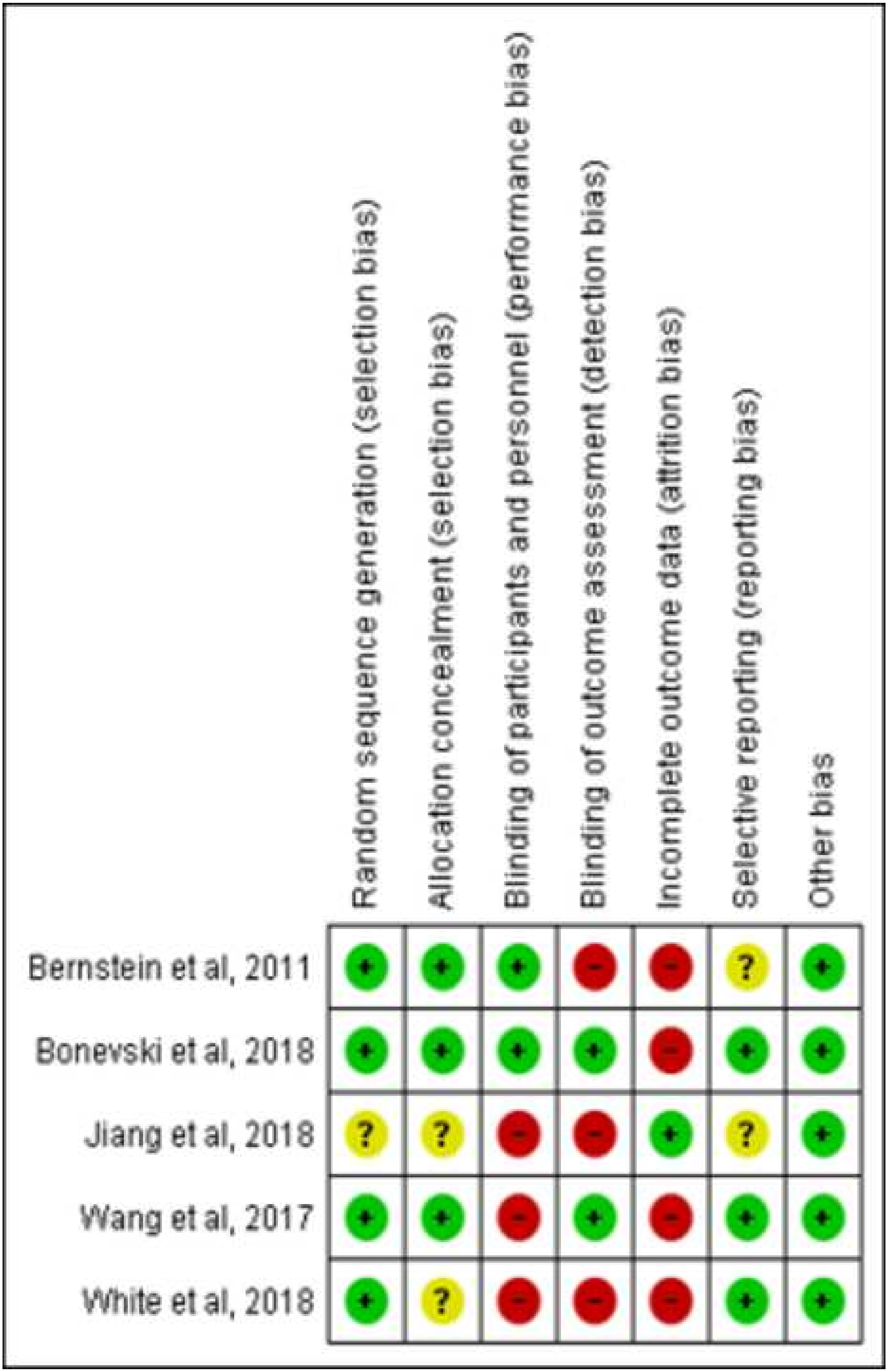

Figure

Click here to access/download;Figure;Figure 3(b).tif

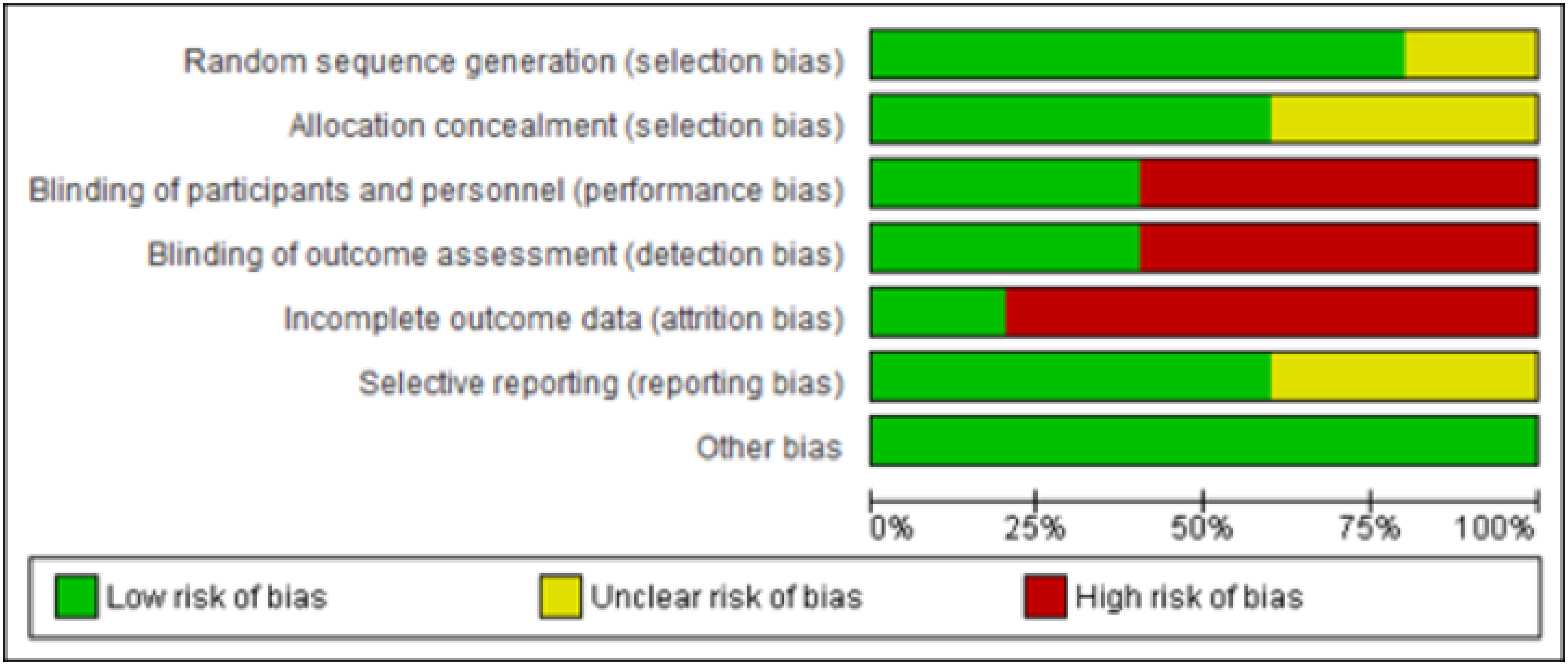

Click here to access/download
Supporting Information
S1 Risk of Bias Assessment.docx

Click here to access/download
Supporting Information
S2 PRISMA Checklist.doc
