# Supplementary material for: Effectiveness of community health workers involvement in smoking cessation programme: A systematic review
Source: PLoS One. 2020 Nov 19;15(11):e0242691. doi: 10.1371/journal.pone.0242691 (PMC7676728; doi:10.1371/journal.pone.0242691)
Supplement: Attachment — Submitted filename: PONE-D-20-06267_R1_02102020.pdf [file pone.0242691.s004.pdf]

PLOS ONE

Effectiveness of Community Health Workers Involvement in Smoking Cessation Programme: A Systematic Review

--Manuscript Draft--

**Manuscript Number:**	PONE-D-20-06267R1
**Article Type:**	Research Article
**Full Title:**	Effectiveness of Community Health Workers Involvement in Smoking Cessation Programme: A Systematic Review
**Short Title:**	Community health workers involvement in smoking cessation
**Corresponding Author:**	Siti Hafizah Zulkiply, M.D<br>Universiti Putra Malaysia<br>MALAYSIA
**Keywords:**	Community health worker; smoking cessation; Sustainable Development Goals; primary care
**Abstract:**	Background: Sustainable Development Goals (SDG) has set the target to reduce premature mortalities from non-communicable diseases (NCDs) by one-third. One of the ways to achieve this is through strengthening the countries' implementation of the World Health Organization Framework Convention on Tobacco Control (WHO FCTC). Community health workers (CHWs) involvement has shown promising results in the prevention of NCDs. This systematic review is aimed at critically evaluating the available evidence on the effectiveness of involving CHWs in smoking cessation.<br><br>Materials and Methods: We systemically searched PubMed and CENTRAL up to September 2019. We searched for published interventional studies on smoking cessation interventions using the usual care that complemented with CHWs as compared to the usual or standard care alone. Our primary outcome was abstinence of smoking. Two reviewers independently extracted data and assessed study risks of bias.<br><br>Result: We identified 2794 articles, of which only five studies were included. A total of 3512 smokers with 41 CHWs were included in the studies. The intervention duration range from 6 weeks to 30 months. The studies used behavioral intervention or a combination of behavioral intervention and pharmacological treatment. Overall, the smoking cessation intervention that incorporated involvement of CHWs had higher smoking cessation rates [OR 1.95, 95% CI (1.35,2.82)]. Significant smoking cessation rates were seen in two studies.<br><br>Conclusion: Higher smoking cessation rates were seen in the interventions that combined the usual care with interventions by CHWs as compared to the usual care alone. However, there were insufficient studies to prove the effectiveness. In addition, there was high heterogeneity in terms of interventions and participants in the current studies.
**Order of Authors:**	Siti Hafizah Zulkiply, M.D
	Lina Farhana Ramli
	Zul Aizat Mohamad Fisal
	Bushra Tabassum
	Rosliza Abdul Manaf
**Response to Reviewers:**	Reviewer 1<br>Results: Figure 2 is not described in the text and also not mentioned in methods. Please include some information about it.<br>Figure 2 is described in line 226 under heading 3.4 Smoking abstinence rate<br><br>Was the review registered in PROSPERO?<br>The review was not registered in PROSPERO

Powered by Editorial Manager® and ProduXion Manager® from Aries Systems Corporation

Can you provide more specifications about what type of studies were included? e.g. clinical trials, clinical answers, etc

Type of studies included is describe in line 125 under eligibility criteria- This study only included intervention studies or controlled clinical trials that compared a combination of interventions given by CHWs and usual care with only usual care.

There is no information about the duplicated data and its exclusion. Was duplicated information founded? If so, the information should also be shown in the PRISMA flow diagram (Results)
Data of duplicates removed is shown in PRISMA flowchart (Figure1) and described in 3.1 search result sections

Data Extraction, Data Analysis and Risk of Bias assessment:
- In terms of effectiveness, more details of the analysis and results (outcomes) should be described
OR of the studies has been included

Conclusions:
- Describe more about the limitations of the review in terms of participants and study designs and also availability of data.
Limitation on the heterogeneity and insufficient study are included

References: In general, the consistency and format of references should be revised: pages, p at the ending, links, etc

Review first reference, pages missing
Reference 1 has been revised

Reference 6: review the reference and format
Reference 6 has been revised

Line 44-46: Reference missing
Reference added for line 44-46

Line 55: Is reference 3 correct?
The reference has been updated

Line 66: "forecasted that more than 8 million people will die from diseases related to tobacco use by year 2030 if pattern of smoking continues" is the reference the same?
Yes, the reference is the same

Line 72-74: "with the highest prevalence of smokers were among 25 to 44 years old age group (28%), followed by 45 to 64 years old age group (20%) (3)." Are these group ages of specific special interest? Because in the non-aggregated data of the original paper there is variation in that age group.
No, its not of specific interest

Line 119-121: Include references
Reference included

Line 176: Reference 73 is not available
73 was referred to number of studies and not references.

Reviewer 2

Need more data on prevalence of smokers in rural and urban is included
95% CI is included

Add details on search for publication years
Details on search for publication years from 2009 to 2019, was added in line 137

Powered by Editorial Manager® and ProduXion Manager® from Aries Systems Corporation

	Details on population type<br>Added specific ethnicity in line 151<br><br>Details on type of intervention<br>Added behavioural or pharmacological in line 152<br><br>Details on validated abstinence rate<br>Added chemically verified using CO in line 163<br><br>Details on irrelevant study designs<br>Added observational studied or interventional studies without control group in line 178<br><br>Details about CHWs, differences by demographics<br>Added level of education in table 3, discuss differences by demographics<br><br>Details on differences of targeted communities with cultural backgrounds<br>Details on differences of targeted communities included<br><br>Details on behavioral interventions<br>Details on behavioral interventions included<br><br>Risk of bias<br>The risk of bias assessment was done according to COCHRANE guideline for assessment of systematic reviews. The details of the risk of bias assessment is presented in S2 Table
**Additional Information:**	
**Question**	**Response**
**Financial Disclosure**<br><br>Enter a financial disclosure statement that describes the sources of funding for the work included in this submission. Review the submission guidelines for detailed requirements. View published research articles from *PLOS ONE* for specific examples.<br><br>This statement is required for submission and **will appear in the published article** if the submission is accepted. Please make sure it is accurate.	Authors received no specific funding

**Unfunded studies**<br>Enter: *The author(s) received no specific funding for this work.*<br><br>**Funded studies**<br>Enter a statement with the following details:<br>• Initials of the authors who received each award<br>• Grant numbers awarded to each author<br>• The full name of each funder<br>• URL of each funder website<br>• Did the sponsors or funders play any role in the study design, data collection and analysis, decision to publish, or preparation of the manuscript?<br>• **NO** - Include this sentence at the end of your statement: *The funders had no role in study design, data collection and analysis, decision to publish, or preparation of the manuscript.*<br>• **YES** - Specify the role(s) played.<br><br>* typeset	
**Competing Interests**<br><br>Use the instructions below to enter a competing interest statement for this submission. On behalf of all authors, disclose any competing interests that could be perceived to bias this work—acknowledging all financial support and any other relevant financial or non-financial competing interests.<br><br>This statement **will appear in the published article** if the submission is accepted. Please make sure it is accurate. View published research articles from *PLOS ONE* for specific examples.	We declared no competing interests

**NO authors have competing interests**<br><br>Enter: *The authors have declared that no competing interests exist.*<br><br>**Authors with competing interests**<br><br>Enter competing interest details beginning with this statement:<br><br>*I have read the journal's policy and the authors of this manuscript have the following competing interests: [insert competing interests here]*<br><br>* typeset	
**Ethics Statement**<br><br>Enter an ethics statement for this submission. This statement is required if the study involved:<br><br>• Human participants<br>• Human specimens or tissue<br>• Vertebrate animals or cephalopods<br>• Vertebrate embryos or tissues<br>• Field research<br><br>Write "N/A" if the submission does not require an ethics statement.<br><br>General guidance is provided below. Consult the submission guidelines for detailed instructions. **Make sure that all information entered here is included in the Methods section of the manuscript.**	N/A

**Format for specific study types**<br><br>**Human Subject Research (involving human participants and/or tissue)**<br>• Give the name of the institutional review board or ethics committee that approved the study<br>• Include the approval number and/or a statement indicating approval of this research<br>• Indicate the form of consent obtained (written/oral) or the reason that consent was not obtained (e.g. the data were analyzed anonymously)<br><br>**Animal Research (involving vertebrate animals, embryos or tissues)**<br>• Provide the name of the Institutional Animal Care and Use Committee (IACUC) or other relevant ethics board that reviewed the study protocol, and indicate whether they approved this research or granted a formal waiver of ethical approval<br>• Include an approval number if one was obtained<br>• If the study involved *non-human primates*, add *additional details* about animal welfare and steps taken to ameliorate suffering<br>• If anesthesia, euthanasia, or any kind of animal sacrifice is part of the study, include briefly which substances and/or methods were applied<br><br>**Field Research**<br><br>Include the following details if this study involves the collection of plant, animal, or other materials from a natural setting:<br>• Field permit number<br>• Name of the institution or relevant body that granted permission	
**Data Availability**<br><br>Authors are required to make all data underlying the findings described fully available, without restriction, and from the time of publication. PLOS allows rare exceptions to address legal and ethical concerns. See the PLOS Data Policy and FAQ for detailed information.	Yes - all data are fully available without restriction

Powered by Editorial Manager® and ProduXion Manager® from Aries Systems Corporation

A Data Availability Statement describing where the data can be found is required at submission. Your answers to this question constitute the Data Availability Statement and **will be published in the article**, if accepted.<br><br>**Important:** Stating 'data available on request from the author' is not sufficient. If your data are only available upon request, select 'No' for the first question and explain your exceptional situation in the text box.<br><br>Do the authors confirm that all data underlying the findings described in their manuscript are fully available without restriction?	
**Describe where the data may be found in full sentences. If you are copying our sample text, replace any instances of XXX with the appropriate details.**<br><br>• If the data are **held or will be held in a public repository**, include URLs, accession numbers or DOIs. If this information will only be available after acceptance, indicate this by ticking the box below. For example: *All XXX files are available from the XXX database (accession number(s) XXX, XXX.*).<br>• If the data are all contained **within the manuscript and/or Supporting Information files**, enter the following: *All relevant data are within the manuscript and its Supporting Information files.*<br>• If neither of these applies but you are able to provide **details of access elsewhere**, with or without limitations, please do so. For example:<br><br>*Data cannot be shared publicly because of [XXX]. Data are available from the XXX Institutional Data Access / Ethics Committee (contact via XXX) for researchers who meet the criteria for access to confidential data.*<br><br>*The data underlying the results presented in the study are available from (include the name of the third party*	all relevant data are within the manuscript and its supporting information files

and contact information or URL).<br>• This text is appropriate if the data are owned by a third party and authors do not have permission to share the data.<br><br>* typeset	
Additional data availability information:	

Manuscript Click here to access/download;Manuscript;Manuscript.docx

Effectiveness of Community Health Workers Involvement in Smoking Cessation Programme: A Systematic Review.

Siti Hafizah Zulkiply*, Lina Farhana Ramli, Zul Aizat Mohamad Fisal, Bushra Tabassum, Rosliza Binti Abdul Manaf*.

Faculty of Medicine and Health Sciences, University Putra Malaysia
*Corresponding authors: Siti Hafizah Binti Zulkiply, hafizahzulkiply.hz@gmail.com
Rosliza Binti Abdul Manaf, rosliza_abmanaf@upm.edu.my

Abstract

Background: Sustainable Development Goals (SDG) has set the target to reduce premature mortalities from non-communicable diseases (NCDs) by one-third. One of the ways to achieve this is through strengthening the countries' implementation of the World Health Organization Framework Convention on Tobacco Control (WHO FCTC). Community health workers (CHWs) involvement has shown promising results in the prevention of NCDs. This systematic review is aimed at critically evaluating the available evidence on the effectiveness of involving CHWs in smoking cessation.

Materials and Methods: We systemically searched PubMed and CENTRAL up to September 2019. We searched for published interventional studies on smoking cessation interventions using the usual care that complemented with CHWs as compared to the usual or standard care alone. Our primary outcome was abstinence of smoking. Two reviewers independently extracted data and assessed study risks of bias.

Result: We identified 2794 articles, of which only five studies were included. A total of 3512 smokers with 41 CHWs were included in the studies. The intervention duration range from 6 weeks to 30 months. The studies used behavioral intervention or a combination of behavioral intervention and pharmacological treatment. Overall, the smoking cessation intervention that incorporated involvement of CHWs had higher smoking cessation rates [OR 1.95, 95% CI (1.35,2.82)]. Significant smoking cessation rates were seen in two studies.

Conclusion: Higher smoking cessation rates were seen in the interventions that combined the usual care with interventions by CHWs as compared to the usual care alone. However, there were insufficient studies to prove the effectiveness. In addition, there was high heterogeneity in terms of interventions and participants in the current studies.

Keywords: Community Health Worker, Smoking Cessation, Sustainable Development Goals, Primary Care

Introduction

Non-communicable diseases (NCD) are the major cause of mortalities worldwide. Smoking is an important risk factor for the development of NCD, including cancers, and cardiovascular and respiratory diseases (1). The United Nation General Assembly has developed an agenda for the Sustainable Developmental Goals (SDG) in 2015, containing a total of 17 Goals that all Member States have agreed to achieve by 2030 (2). SDG 3, with goal the to “ensure healthy lives and promote well-being for all ages” includes target 3.4, which is to reduce premature mortality from NCD by one-third, and target 3a, which is to strengthen a country’s implementation of the World Health Organization Framework Convention on Tobacco Control (WHO FCTC) (2,3).

In September 2005, Malaysia had participated with WHO FCTC and agreed to fulfil the demand to develop and disseminate a standard national guideline on tobacco (4). In response, government of Malaysia (GoM) had set up a target to reduce 30% relative reduction of national smoking prevalence by 2025 within the National Strategic Plan for NCD (NSP-NCD). In addition, the World Health Organization (WHO) has also developed MPOWER that include measures for demand reduction: i) Monitoring tobacco use and prevention policies; ii) Protecting people from tobacco smoke; iii) Offering help to quit tobacco use; iv) Warning about the dangers of tobacco; v) Enforcing bans on tobacco advertising, promotion and sponsorship; and vi) Raising taxes on tobacco (5).

Tobacco has become a major public health enemy, accountable for more than 7 million preventable deaths yearly, and it has been forecasted that more than 8 million people will die from diseases related to tobacco use by year 2030 if pattern of smoking continues (6). In Malaysia alone, it is estimated that more than 20,000 have died due to tobacco each year (7). There are 933.1 million smokers worldwide in 2015 and around 80% of them live in low and

middle-income countries (LMICs) (World Health Organization Media Center, 2017). According to the National Health and Morbidity Survey (NHMS) 2015, the prevalence of smokers in Malaysia was 22.8%, therefore, it is estimated that nearly five million Malaysians aged 15 years and above are smokers (4). The mean age of smoking initiation is 18.3 years (9), with the highest prevalence of smokers being among the 25 to 44-years-old age group (28%), followed by the 45 to 64-years-old age group (20%) (4).

In many countries, smoking cessation programs (identification of smokers, advising and offering support to quit) are taking place in primary care settings (10). Evidence for the effectiveness of these interventions in this setting administered by professional healthcare providers is well established (11). Since 2004, Malaysia has set up quit-smoking clinics available at most primary health clinics (12). Unfortunately, studies have shown that high mortalities in low socioeconomic areas, especially secondary to NCD are due to barriers in healthcare services (13). Since the low socioeconomic status is an important determinant of smoking (14), it is pertinent that interventions on smoking should focus on these populations. As reported by Global Adult Tobacco Survey (GATS) 2011, the prevalence of smokers in rural areas was higher (24.3%, 95% CI 22.0, 26.7) as compared to urban area (22.7%, 95% CI 20.2, 25.4) (15). The WHO has developed an effective community-based strategy to reduce the gap in healthcare in low-socioeconomic areas. However, the current smoking cessation services are not user-friendly and poorly understood (16).

In line with the Alma Ata declaration of the Primary Healthcare Concept, all community health strategy designs must address the community needs at the local level, be led by the community members themselves, and require involvement of communities to mobilize local resources. Task shifting, an idea conceived by the WHO, is defined as redistributing primary

care responsibilities from physician to non-physician providers (17). Task shifting can be further extended to include health workers without formal healthcare training, known as community health workers (CHWs) (18). In 1989, a WHO study group had established a widely accepted definition of CHWs: the workers should be among the communities where they practice, be chosen by the communities, be able to answer to the public for their programs, be supported by the health system, and have less duration of training than professional workers (19). CHWs have the potential to achieve primary health care goals, by enhancing access to care and promote a proper use of health resources through the provision of cultural and outreach links between communities and health systems, giving health education to reduce cost; and, screening services, basic emergency services, continuum of care and client protection (19). Furthermore, it is imperative to enhance civic engagement and ensure accountability in order to achieve the SDG goals (20,21). There are considerable evidences supporting positive impacts of CHWs on the health of diverse populations especially on the maternal and child health, malaria and tuberculosis. Numerous studies have also evaluated the role of community intervention on major cardiovascular events and risk factors. Community-based cardiovascular health intervention in vulnerable populations has shown that the interventions aimed at decreasing blood pressure are the most promising and behavior change interventions are the most challenging (22,23). Task-shifting interventions were also proven to be effective in lowering the low-density lipoprotein cholesterol (LDL) and total cholesterol (24). In another review, significant reduction in the mean blood pressure and glycated haemoglobin levels were seen in task-shifting interventions for the CVD risk reduction in LMICs (24,25).

Guidelines on smoking cessation programs have shown that the combinations of behavioral change and pharmacological support given by the professional healthcare providers are the

most effective. CHWs are crucial as they serve as a critical link in increasing the communities' access to services, especially for people living in rural and undeserved areas, which ultimately form an integral part needed to achieve the SDG goals. Therefore, the aim of this study is to evaluate the effectiveness of involving CHWs in smoking cessation programs as compared to the usual care.

Materials and Methods

This review was conducted and reported in accordance to the Preferred Reporting Items for Systematic Reviews and Meta-Analyses (PRISMA) guidelines and based on the Cochrane Collaboration approach (26,27).

2.1 Eligibility Criteria

We included intervention studies or controlled clinical trials that compared between a combination of interventions given by CHWs and usual care, and only usual care. Only interventional studies conducted among smokers aged 18 years old and above, having the smoking abstinence rate as the outcome were included. We excluded studies that conducted intervention in adolescent or special group (defines as mental illness or LGBTQ). Non-English language studies, reviews, proceedings, qualitative studies, descriptive studies and protocol were also excluded.

2.2 Data Sources and Search Strategy

We systematically searched for relevant articles published in The Cochrane Central Register of Controlled Trials (CENTRAL) and PubMed/MEDLINE up to September 2019. The search was limited to 10 years, from 2009 until 2019. We combined the keywords for smoking,

cessation and community as the following: cessation or reduction AND smoking or tobacco AND volunteer or peer or community or lay.

2.3 Study Selection

A pair of authors independently assessed the titles and abstracts of a defined set of articles. Each study was recorded as include, exclude or unclear. The full articles were retrieved for further assessment if they were recorded as include or unclear. Eligible studies were identified based on the inclusion criteria. Any discrepancies in the assessment were resolved by discussion leading to a consensus.

2.4 Data Extraction, Data Analysis and Risk of Bias Assessment

Data extraction from all potential studies was documented in a table. The table included information on study characteristics (sample size and study duration), participant characteristics (setting, population type, and specific ethnicity), intervention characteristics (type of interventions given in both arms, and behavioural or pharmacological intervention), training of CHWs (background, duration of training and training module) and analysis and results (outcomes) of both arms. All authors independently extracted the data and any discrepancies were resolved by discussion. The characteristics of the included studies are outlined in Table 1 through Table 3.

Data synthesis and analysis were carried out using Review Manager Software (Rev Man) version 5.3 (Nordic Cochrane Centre, Cochrane Collaboration, Copenhagen). The end of study values were taken. If studies measured smoking cessation rates at multiple intervals (3 or 6 or 9 months), the outcomes in the final point of interval were taken. If studies reported

two types of smoking cessation measurements (self-reported or chemically verified using carbon monoxide (CO) level), the chemically verified outcomes were taken.

We assessed the study quality of each study using the COCHRANE guideline for assessment of systematic reviews and the published assessment guide on risks of bias assessment for intervention studies (26). The studies were evaluated based on eight criteria: randomized treatment order, allocation concealment, blinding of participants and personnel, blinding of outcome assessment, incomplete outcome data, selective outcome reporting and other bias. For each item, risk of bias was classified as 'low risk', 'high risk, or 'unclear risk', with the last category indicating either a lack of information or uncertainty over the potential for bias. The results were presented in a 'Risk of bias' summary (Figure 3a and 3b) and S2 Table.

Result

3.1 Search Results

We identified 2794 articles through our electronic database search. After excluding 280 duplicated studies, a total of 2614 articles were excluded following titles and abstracts screening due to irrelevant study designs (observational studies or interventional studies without control group), irrelevant outcomes (no smoking cessation or did not provide smoking abstinence rate), and irrelevant interventions (not given by CHWs). One hundred full text articles were screened, of which 95 articles were excluded for following reasons: interventions were not given by CHWs (73 studies), study designs were non controlled trials (17 studies) and outcomes were not smoking cessation (5 studies). Finally, five studies were included in this review (28–32). Figure 1 shows PRISMA flowchart.

Figure 1: PRISMA flowchart for the selection of studies. Outcomes of the systematic review of the literature by record identification, screening, and analysis in the Preferred Reporting Items for Systematic Reviews and Meta-Analyses (PRISMA) statement flow diagram.

3.2 Characteristics of Included Studies

A total of 3512 participants were included in this review. The studies were conducted between the year of 2011 and 2018. Two studies were conducted in United States of America (USA) (28,29) while the other studies were conducted in Hong Kong (30), Australia (32) and Vietnam (31). The duration of the studies conducted ranged from 6 weeks to 30 months. All studies were conducted among the community, except for one study conducted in an emergency department setting (28). The same study was conducted on a specific ethnicities (African American and Hispanic community) (28). There were no significant differences between the baseline number of cigarettes per day (13.08 ± 9.57).

Table 1. Characteristics of the studies

Study	Setting	Participants type	Sample size	Intervention	Control	Mean cigarettes/ day	Study Design	Study Duration
**Bernstein *et al*, 2011** (28)	Bronx, New York, United States of America	Hispanic and African American	337	170	167	15 ± 7.48	RCT	21 months
**Wang *et al*, 2017** (30)	Hong Kong	Public	1,226	402	416<br>408	N/A	Cluster-3 arms RCT	3 months
**White *et al*, 2018** (29)	Maryland, United States of America	Public	200	101	99	19.3 ± 18.3	RCT	6 – 8 weeks
**Bonevski *et al*, 2018** (32)	New South Wales, Australia	Disadvantage Adult clients of the Community Care Centre	431	187	244	15 ± 7.46	Parallel randomised trial	30 months
**Jiang *et al*, 2018** (31)	Thai Nguyen, Vietnam	Village Population	1,318	781	537	11.02 ± 9.48	RCT and Quasi-experimental	6 months

Abbreviations: RCT- randomized controlled trial

3.3 Characteristic of Intervention

All of the studies used behavioral intervention such as brief advice as the method for smoking cessation. The usual care intervention given to both intervention groups and control group were mainly brief advice, and some of the studies provided self-help material. Four studies used brief advice; one study used AWARD model (Ask, Warn, Advise, Refer and Do-it-again) (30), one study used 4A's model (Ask for tobacco use, Advise to quit, Assess readiness to quit, and Assist) (31), one study used automated Smokefree TXT (29) and one study used on-screen advice (32). Self-help materials in the form of leaflet or brochure were provided to the participants in three studies (28,30,31).

The interventions given by CHWs in the studies were behavioral intervention, active referral and pharmacological therapy. Two studies used motivational interviewing (MI), and provided pharmacological treatment (nicotine replacement therapy (NRT)) to the participants (28,32). One study had active referral to the existing smoking cessation intervention in clinics by CHWs (30). One study used personalized text messages (29) as a mediator for smoking cessation intervention. Table 2 shows the details of the interventions.

3.4 Smoking Abstinence Rate

All studies used intention to treat analysis, except for the study by Bonevski et al. (32). The outcomes of the smoking abstinence rate were measured by either self-reporting or chemically verified using CO level. Two studies provided abstinence rate after 3 months (28,29) and the remaining three studies after 6 months (30–32).

The pooled OR were estimated using random effects models as the studies included were heterogenous with respect to interventions and populations. Overall, all studies had higher

odds of abstinence in the intervention group as compared to the usual group [OR 1.95, 95% CI (1.35,2.82)]. Two studies reported significant smoking abstinence rate in the interventions using CHWs, with OR 2.96 [2.15, 4.07] ($p<0.001$) (31), OR 1.81 [1.04, 3.16] (active referral vs brief advice group), and OR 1.85 [1.06, 3.23] (active referral vs control group) (30). Figure 2 and Table 2 show the details of the result.

Figure 2: Forest plot shows smoking abstinence rate.

Table 2. Characteristic of Intervention and Outcome

Study	Intervention	Control	Outcome Intervention	Outcome Control
**Bernstein *et al*, 2011** (28)	Smoking cessation brochure (10-15 min) AND MI by interventionist, 6 weeks course of NRT.	Smoking cessation brochure and contact information to smoking cessation programs	Self-reported 7-days PPA rates at 3 months: 14.7%<br>OR 1.14 [0.61, 2.11]	Self-reported 7-days PPA rates at three months: 13.2%
**Wang *et al*, 2017** (30)	Brief advice using structural model AWARD, health warning leaflet AND active referral to SC services by ambassadors	Brief advice using structural model AWARD, health-warning leaflet and encourage to SC services	Validated abstinence rates at 6 months: 9.0% ($p<0.05$)<br>OR 1.81 [1.04, 3.16]<br>OR 1.85 [1.06, 3.23]	Validated abstinence rates at 6 months:<br>5.0% and<br>5.1%
**White *et al*, 2018** (29)	Automated SFTXT<br>AND personalized text messages from peer mentor	Automated SFTXT messages	Self-reported 7-day PPA rates at 3 months: 7.9%<br>OR 2.75 [0.71, 10.70]	Self-reported 7-day PPA rates at 3 months: 3.0%
**Bonevski *et al*, 2018** (32)	On screen advice to quit smoking, state Quitline telephone number, and a gift bag with call it quits<br>AND MI and NRT by trainer volunteer case workers	On screen advice to quit smoking, state Quitline telephone number, and a gift bag with call it quits	Self-reported continuous verified PPA at 6 months: 1.0%<br>OR 0.76 [0.07, 8.44]	Self-reported continuous verified PPA at 6 months: 1.4%
**Jiang *et al*, 2018** (31)	4As (brief counselling and educational materials) AND refer smokers to a trained VHW (4As+R)	4As (brief counselling and educational materials)	Validated abstinence rate at 6 months: 25.7% ($p<0.001$)<br>OR 2.96 [2.15, 4.07]	Validated abstinence rate at 6 months: 10.5%

Abbreviations: MI- Motivational Interviewing; NRT- nicotine replacement therapy; PPA- point prevalence abstinence; SC- smoking cessation; CHW-Community Health Worker; AWARD; SFTXT- SmokefreeTXT; SC- smoking cessation; 4As- Ask (screen for tobacco use), Advise to quit, Assess readiness to quit, and Assist; VHW- Village Health Worker.

3.5 Characteristics and Training of Community Health Workers (CHWs)

The number of CHWs in the study varied from one person to 32 persons; two studies did not provide the amount of CHWs (30,32). Only one study had CHWs from the same community with the participants (31). Two studies had volunteers from NGOs (30,32) or university students (30). CHWs from two studies had prior experiences working as CHWs or worked on smoking cessation programs previously (29,31). Two studies had CHWs who were former smokers (28,29).

The duration of training of the CHWs ranged from just two hours to two weeks. The training were given by either the researchers or experts in the field. The content of the training was comprised of the study protocol and method in smoking cessation intervention. Supervisions were given weekly or biweekly to ensure fidelity. Only one study provided incentives to the CHWs (29). Table 3 shows the details of the characteristics of CHWs and their training.

Table 3: Characteristics of CHWs and training of CHWs.

Table 3. Characteristic of Community Health Worker and Training of Community Health Worker

Study	Amount and Description	Educational Background	Experience	Incentive	Duration/ Training Module/ Supervision
**Bernstein *et al*, 2011** (28)	One (Interventionist)<br>Former smoker<br>ED based	Not reported	Not reported	None	Duration: 2 weeks<br><br>Training module: Course and practicum (roleplay, slide shows and observation)- Epidemiology, health effects and treatment of tobacco dependence, motivational interviewing.<br><br>Supervision: Every two weeks
**Wang *et al*, 2017** (30)	Not mentioned (SC ambassador)	University students (health-related studies)*.	Volunteers from NGO*.	None	Duration: 4 hours<br><br>Training module: Tobacco control and SC, SC reduction advice skills<br><br>Supervision: Spot checks
**White et al, 2018** (29)	Thirty-two (Peer mentor)<br>Former smoker more than 1 year<br>18 years old and above<br>Lived in the United States<br>Willingness to mentor smokers through texting and completing the online training program	Not reported	Facilitators of the American Cancer Society Freshstart group-based cessation support program*.	$200 and entry into $1000 drawing	Duration: 2 hours<br><br>Training module: Online- study details, smoking and SC, MI, web-based-text-messaging platform<br><br>Supervision: N/A
**Bonevski et al, 2018** (32)	Not mentioned (Caseworker)	Not reported	Volunteer case workers of community social	None	Duration: One day<br><br>Training module: Behavioral counselling and MI

Study	Amount and Description	Educational Background	Experience	Incentive	Duration/ Training Module/ Supervision
			service organizations (NGO) in NSW.		Supervision: N/A
**Jiang et al, 2018** (31)	Six to eight (VHW)<br>Not a current smoker and willing to participate in the required components of the study intervention.	Not reported	Worked as a VHW at that particular site for a year or more.	None	Duration: 4 days<br><br>Training module: Research ethics, study protocol, use of CO monitor, theory of behavior change, MI, social cognitive skills building approach.<br><br>Supervision: N/A

Abbreviations: N/A- not available; SC-Smoking Cessation; MI-motivational Interviewing; ED- Emergency Department; NGO-non-governmental organizations; NSW-New South Wales; VHW-village health worker.

*the paper did not mentioned details of the experience.

3.6 Risk of Bias Assessment

We judged the risk of possible bias present in the studies according to the four incorporated criteria. We presented the summary of risk of bias assessment of the studies in S2 Table and Figure 3(a) to 3(b). The random sequence generation was assessed to be of low risk of bias in all studies except for the study by Jiang et al. (31) as they did not clearly state the method of random sequence generation. When assessing the allocation concealment of the included studies, three studies (28,30,32) had low risk of bias while the other two studies (29,31) did not clearly state the method of allocating the treatment groups. Performance bias was assessed to be low risk in only two studies (28,32) with the remaining studies assessed to be high risk. The detection bias was assessed to be low risk in two studies (30,32). Only the study by Jiang et al. had low risk in attrition bias (31) while the remaining studies were assessed to be high risk due to the high dropout rate (>20%). Three studies with available protocol and all the pre-specified outcomes measured were considered as low risk of reporting bias (29,30,32).

Figure 3 (a) and 3 (b): Risk of bias assessment.

Discussion

This review evaluated the effectiveness of involving CHWs in smoking cessation programs as compared to the usual care. Our review found higher smoking cessation rates in the interventions involving CHWs as compared to usual care in five studies. Evidence of effectiveness of CHWs in the cardiovascular diseases (CVD) management and prevention is still lacking. Previous studies reported evidences on the four main risk of CVDs: hypertension, diabetes mellitus, unhealthy diet and alcohol and tobacco consumption. A study by Jeet et al. reported an increase in tobacco cessation in interventions using CHWs as compared with the standard care (33), as with this review.

The objective of having CHWs is having the intervention given by someone from the community itself. Its effectiveness vary depending on their training program, demographics and settings (34). Significant smoking cessation rates were seen in two studies that conducted the training in 4 hours (30) and 4 days (31). Only one study had CHWs from the same community with the participants, and reported significant reduction of smoking cessation rate. In addition, the study only takes CHWs that have prior experience working as CHWs at that particular site. The performance of CHWs is also associated with their age, skill and educational level (35). Another study that reported significantly higher cessation rate had university students or volunteers from NGOs as the CHWs (30).

The WHO has formulated a set of recommendations that provides guidance for the task-shifting approaches. These recommendations have implications for a range of health services including the management of NCDs. The main enablers of CHWs interventions are provision of algorithms and protocols, while, restrictions on prescribing medications and availability of medicines are the main barriers identified. Therefore, to ensure its effectiveness, a sound protocol should be developed. However, there was a high heterogeneity in the interventions

given among the included studies. The guidelines on smoking cessation programs showed that a combination of behavioral change and pharmacological support are the most effective. All of the studies used behavioural intervention as the method for smoking cessation with two studies providing pharmacological treatment (nicotine replacement therapy) to the participants. Our review showed that significant higher abstinence rates were seen in one study that used active referral, AWARD and leaflet (30) and one study that used 4A's (31) as the method for behavioural intervention.

CHWs productivity is also largely determined by the condition under which they work. The focus on the provision of an enabling work environment for CHWs is essential for achieving high levels of productivity. Jaskiewicz et al. presented a model in which the work environment encompassed four essential elements: workload, supportive supervision, supplies and equipment, and respect from the community and the health system (36). Similarly, important determinant of positive CHWs interventions is community embeddedness, meaning that community members have a sense of ownership of the program (37).

There are several limitations in this review. First is the high heterogeneity in baseline characteristics of the participants and study designs. Theoretical health belief models assert that a person who is in contemplation stage may be easily influenced to receive intervention. Therefore, the studies with the participants of low exposure to smoking and of intention to quit (theoretical health belief models) might be attributed to higher cessation rates. One study that selected participants who already had the intention to quit smoking showed similar results (29). Similarly, one study that included participants with low amount of cigarettes used (one in the past 3 months or 1 months) showed significant smoking cessation rates (30). Meanwhile, one study conducted in a hospital setting (emergency department) reported higher

abstinence rate in smokers who had smoking-related health issues (28). The review of 33 studies reported high-certainty evidence that incentives improve smoking cessation rates (38). Our review showed similar result, whereby one study that provided incentives to the participants had significant smoking cessation rates (30).

Higher retention is significantly associated with higher quit rates. However, there were high attrition rates (loss of follow up of >20%) in most of the studies included in this review, with the highest attrition rates of 42% (32). The reviews reported that increasing age, higher level of education and higher motivation to quit were associated with higher retention (39).

Recommendations

Previous review has reported cost-effectiveness of CHWs interventions as compared to standard care particularly in tuberculosis, malaria and maternal and child health (40,41). We recommend future studies to analyse the cost-effectiveness of using CHWs in smoking cessation programs. Since the effectiveness of CHWs intervention is largely dependent on the framework and training module, it is imperative that the guidelines on the framework are drawn up. We also recommend studies to measure abstinence rates of smoking according to the gold standard.

Conclusion

CHWs have the potential to bridge between primary healthcare providers and communities, and consequently reducing the gap, especially among low socioeconomic populations as recommended by the SDG. Our review reported positive outcomes on using a combination of CHWs and usual care in smoking cessation as compared with usual care alone, however the evidences are insufficient and have high heterogeneity.

// Acknowledgement

We would like to thank the Director General of Health Malaysia, Director of Institute for Medical Research for the permission to publish this article.

Declaration

The authors declare no conflict of interest.

// Funding

The authors received no specific funding.

Authors Contribution

Siti Hafizah Zulkiply: Analysis, Writing- Original draft and final editing.

Lina Farhana Ramli: Analysis- Writing - Original draft

Zul Aizat Mohamad Fisal: Writing- Original draft

Bushra Tabassum: Analysis, Writing- Original draft

Rosliza Abdul Manaf: Concept development and final editing.

References

1. Glantz S, Gonzalez M. Effective tobacco control is key to rapid progress in reduction of non-communicable diseases. The Lancet. 2012.
2. United Nation. Transforming Our World: The 2030 Agenda for Sustainable Development. In: A New Era in Global Health. 2015.
3. World Health Organisation. Who Framework Convention. WHO Framew Conv Tob Control [Internet]. 2003;42. Available from: http://www.who.int/tobacco/framework/WHO_FCTC_english.pdf
4. Institute for Public Health. National Health and Morbidity Survey 2015 (NHMS 2015). Ministry of Health Malaysia. 2015.
5. World Health Organization. MPOWER : Six policies to reverse the tobacco epidemic. WHO Rep Glob Tob Epidemic. 2008. p 23–41.
6. WHO. Monitoring tobacco use and prevention policies fresh and alive. 2017. p 1–135.
7. Ministry of Health Malaysia. Clinical Practice Guidelines on Treatment of Tobacco Use Disorder. 2016.
8. World Health Organization Media Center. WHO Fact Sheet | Tobacco. World Health organization Fact Sheet. 2017.
9. Lim HK, Ghazali SM, Kee CC, Lim KK, Chan YY, Teh HC, et al. Epidemiology of smoking among Malaysian adult males: Prevalence and associated factors. BMC Public Health. 2013;13(1):2–11.
10. Piné-Abata H, Mcneill A, Murray R, Bitton A, Rigotti N, Raw M. A survey of tobacco dependence treatment services in 121 countries. Addiction. 2013;108(8):1476–84.
11. Stead LF, Buitrago D, Preciado N, Sanchez G, Hartmann-Boyce J, Lancaster T. Physician advice for smoking cessation. Cochrane Database of Systematic Reviews. 2013.
12. Fai SC, Yen GK, Malik N. Quit rates at 6 months in a pharmacist-led smoking cessation service in Malaysia. Can Pharm J. 2016 Sep;149(5):303–12.
13. Yusuf S. Improving worldwide access to inexpensive and effective treatments for common cardiovascular diseases. Eur Hear Journal, Suppl. 2018;20:C18–22.
14. Hiscock R, Dobbie F, Bauld L. Smoking cessation and socioeconomic status: An update of existing evidence from a national evaluation of english stop smoking services. Biomed Res Int. 2015;2015.
15. Mohd Yusoff MF, Guat Hiong HT, Singh S, Kuang Hock L. Report of the global adult tobacco survey (GATS). Inst Public Heal. 2011;204.
16. Chean KY, Goh LG, Liew KW, Tan CC, Choi XL, Tan KC, et al. Barriers to smoking cessation: A qualitative study from the perspective of primary care in Malaysia. BMJ Open. 2019;9(7):1–8.
17. World Health Organization. Task Shifting- Global Recommendations & Guidelines. World Heal Organ. 2008;
18. Fulton BD, Scheffler RM, Sparkes SP, Auh EY. Human Resources for Health Health workforce skill mix and task shifting in low. Hum Resour Health. 2011;1–11.
19. Lehmann U, Sanders D. Community health workers: What do we know about them? 2007.
20. Buse K, Hawkes S. Health in the sustainable development goals: Ready for a paradigm shift? Global Health. 2015;11(1):1–8.
21. Joshi R, Peiris D. Task-sharing for the prevention and control of non-communicable diseases. Lancet Glob Heal [Internet]. 2019;7(6):e686–7. Available from: http://dx.doi.org/10.1016/S2214-109X(19)30161-5
22. Joshi R, Thrift AG, Smith C, Praveen D, Vedanthan R, Gyamfi J, et al. Task-shifting for cardiovascular risk factor management: Lessons from the Global Alliance for Chronic Diseases. BMJ Glob Heal. 2018;3:1–8.
23. Anand TN, Joseph LM, Geetha A V., Prabhakaran D, Jeemon P. Task sharing with non-physician health-care workers for management of blood pressure in low-income and middle-income countries: a systematic review and meta-analysis. Lancet Glob Heal [Internet]. 2019;7(6):e761–71. Available from: http://dx.doi.org/10.1016/S2214-109X(19)30077-4

24. Anand TN, Joseph LM, Geetha A V., Chowdhury J, Prabhakaran D, Jeemon P. Task-sharing interventions for cardiovascular risk reduction and lipid outcomes in low- and middle-income countries: A systematic review and meta-analysis. J Clin Lipidol. 2018;12(3):626–42.
25. Ogedegbe G, Gyamfi J, Plange-Rhule J, Surkis A, Rosenthal DM, Airhihenbuwa C, et al. Task shifting interventions for cardiovascular risk reduction in low-income and middle-income countries: A systematic review of randomised controlled trials. BMJ Open. 2014;4(10).
26. Higgins JPT GS (editors). Cochrane Handbook for Systematic Reviews of Interventions Version 5.1.0 . The Cochrane Collaboration . 2011.
27. Liberati A, Altman DG, Tetzlaff J, Mulrow C, Gøtzsche PC, Ioannidis JPA, et al. The PRISMA statement for reporting systematic reviews and meta-analyses of studies that evaluate health care interventions: explanation and elaboration. In: Journal of clinical epidemiology. 2009.
28. Bernstein SL, Bijur P, Cooperman N, Jearld S, Arnsten JH, Moadel A, et al. A randomized trial of a multicomponent cessation strategy for emergency department smokers. Acad Emerg Med. 2011;
29. White JS, Toussaert S, Thrul J, Bontemps-Jones J, Abroms L, Westmaas JL. Peer Mentoring and Automated Text Messages for Smoking Cessation: A Randomized Pilot Trial. Nicotine Tob Res. 2019;1–10.
30. Wang MP, Suen YN, Li WHC, Lam COB, Wu SY Da, Kwong ACS, et al. Intervention with brief cessation advice plus active referral for proactively recruited community smokers: A pragmatic cluster randomized clinical trial. JAMA Intern Med. 2017;177(12):1790–7.
31. Jiang N, Siman N, Cleland CM, Van Devanter N, Nguyen T, Nguyen N, et al. Effectiveness of Village Health Worker–Delivered Smoking Cessation Counseling in Vietnam. Nicotine Tob Res. 2018;1–7.
32. Bonevski B, Twyman L, Paul C, D'Este C, West R, Siahpush M, et al. Smoking cessation intervention delivered by social service organisations for a diverse population of Australian disadvantaged smokers: A pragmatic randomised controlled trial. Prev Med (Baltim) [Internet]. 2018;112(November 2017):38–44. Available from: https://doi.org/10.1016/j.ypmed.2018.04.005
33. Jeet G, Thakur JS, Prinja S, Singh M. Community health workers for noncommunicable diseases prevention and control in developing countries: Evidence and implications. PLoS One [Internet]. 2017;12(7):1–21. Available from: http://dx.doi.org/10.1371/journal.pone.0180640
34. Abdel-All M, Putica B, Praveen D, Abimbola S, Joshi R. Effectiveness of community health worker training programmes for cardiovascular disease management in low-income and middle-income countries: A systematic review. BMJ Open. 2017;7(11):1–11.
35. Chung MHL, Hazmi H, Cheah WL. Role performance of community health volunteers and its associated factors in Kuching district, Sarawak. J Environ Public Health. 2017;2017.
36. Jaskiewicz W, Tulenko K. Increasing community health worker productivity and effectiveness: A review of the influence of the work environment. Hum Resour Health. 2012;10:1–9.
37. Scott K, Beckham SW, Gross M, Pariyo G, Rao KD, Cometto G, et al. What do we know about community-based health worker programs? A systematic review of existing reviews on community health workers. Hum Resour Health. 2018;16(1):1–17.
38. Notley C, Gentry S, Livingstone-Banks J, Bauld L, Perera R, Hartmann-Boyce J. Incentives for smoking cessation. Cochrane Database of Systematic Reviews. 2019.
39. Courtney RJ, Clare P, Boland V, Martire KA, Bonevski B, Hall W, et al. Predictors of retention in a randomised trial of smoking cessation in low-socioeconomic status Australian smokers. Addict Behav [Internet]. 2017;64:13–20. Available from: http://dx.doi.org/10.1016/j.addbeh.2016.07.019
40. Nkonki L, Tugendhaft A, Hofman K. A systematic review of economic evaluations of CHW interventions aimed at improving child health outcomes. Hum Resour Health. 2017;15(1):1–19.
41. Vaughan K, Kok MC, Witter S, Dieleman M. Costs and cost-effectiveness of community health workers: Evidence from a literature review. Hum Resour Health. 2015;13(1).

Supporting Information

S1 PRISMA Checklist

S2 Risk of Bias Details

 Click here to access/download;Figure;Figure-1.tif

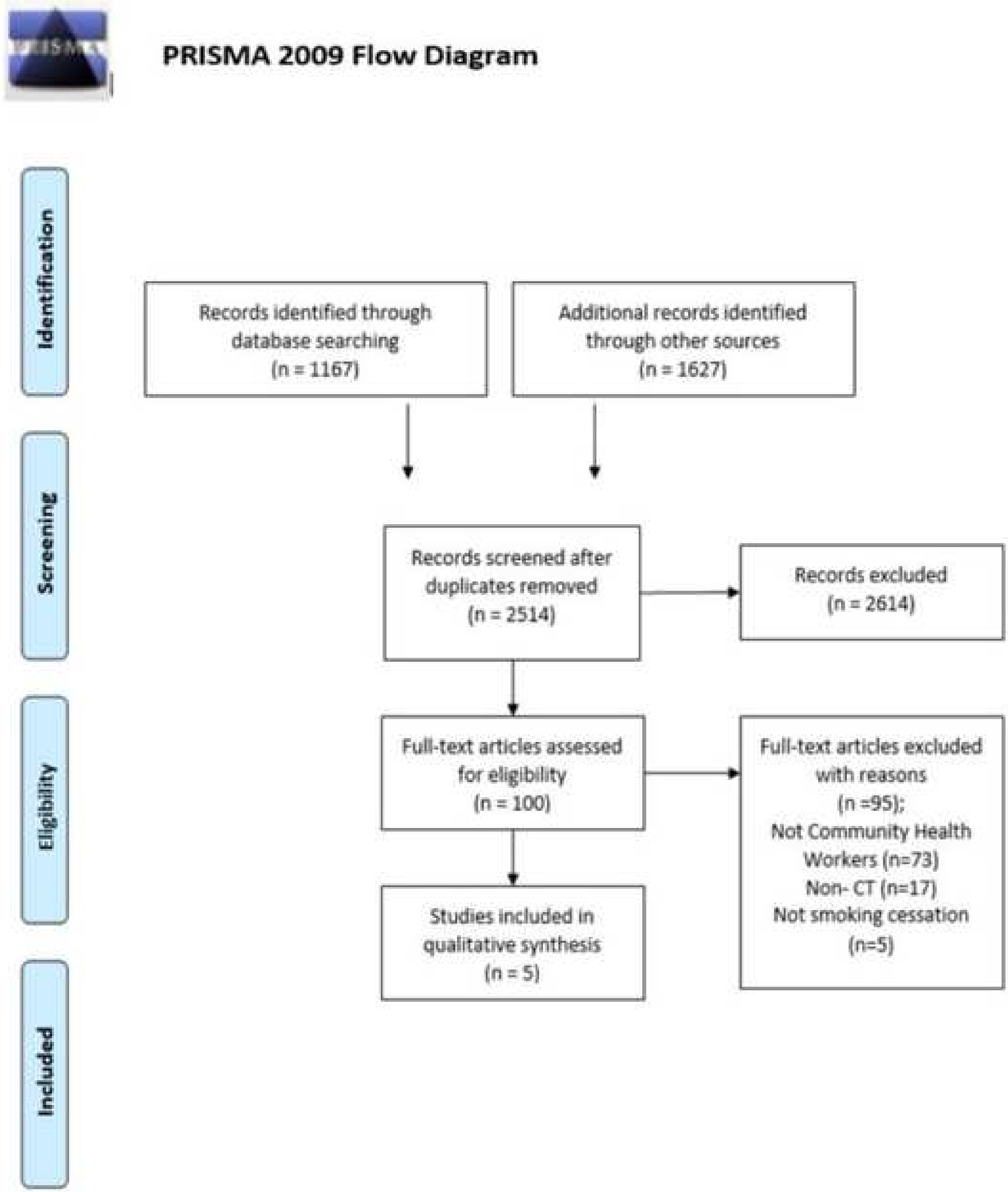

Figure
Click here to access/download;Figure;Figure-2.tif

Study or Subgroup	CHW Events	CHW Total	Control Events	Control Total	Weight	Odds Ratio M-H, Random, 95% CI
Bernstein et al, 2011	25	170	22	167	18.8%	1.14 [0.61, 2.11]
Bonevski et al, 2018	1	99	2	150	2.2%	0.76 [0.07, 8.44]
Jiang et al, 2018	200	781	56	537	30.9%	2.96 [2.15, 4.07]
Wang et al, 2017	36	402	21	416	20.9%	1.85 [1.06, 3.23]
Wang et al, 2017	36	402	21	408	20.9%	1.81 [1.04, 3.16]
White et al, 2018	8	101	3	99	6.2%	2.75 [0.71, 10.70]
**Total (95% CI)**		**1955**		**1777**	**100.0%**	**1.95 [1.35, 2.82]**
Total events	306		125			

Heterogeneity: $Tau^2 = 0.09$; $Chi^2 = 9.39$, $df = 5$ ($P = 0.09$); $I^2 = 47\%$

Test for overall effect: $Z = 3.58$ ($P = 0.0003$)

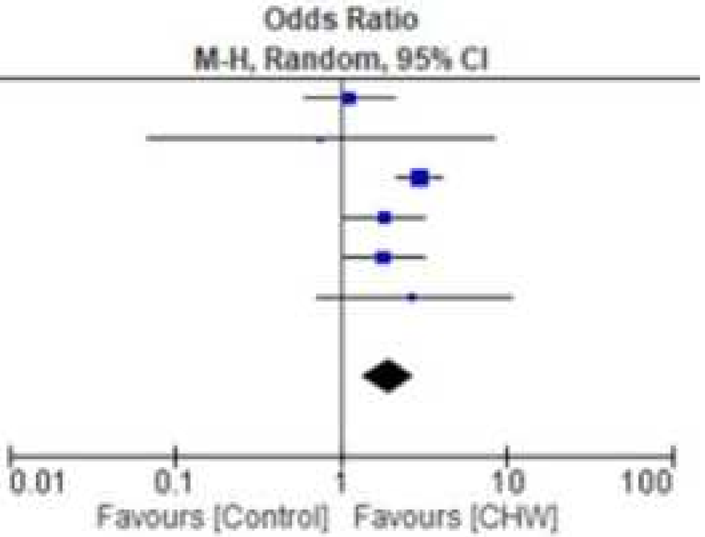

Figure

Click here to access/download;Figure;Figure-3a.tif

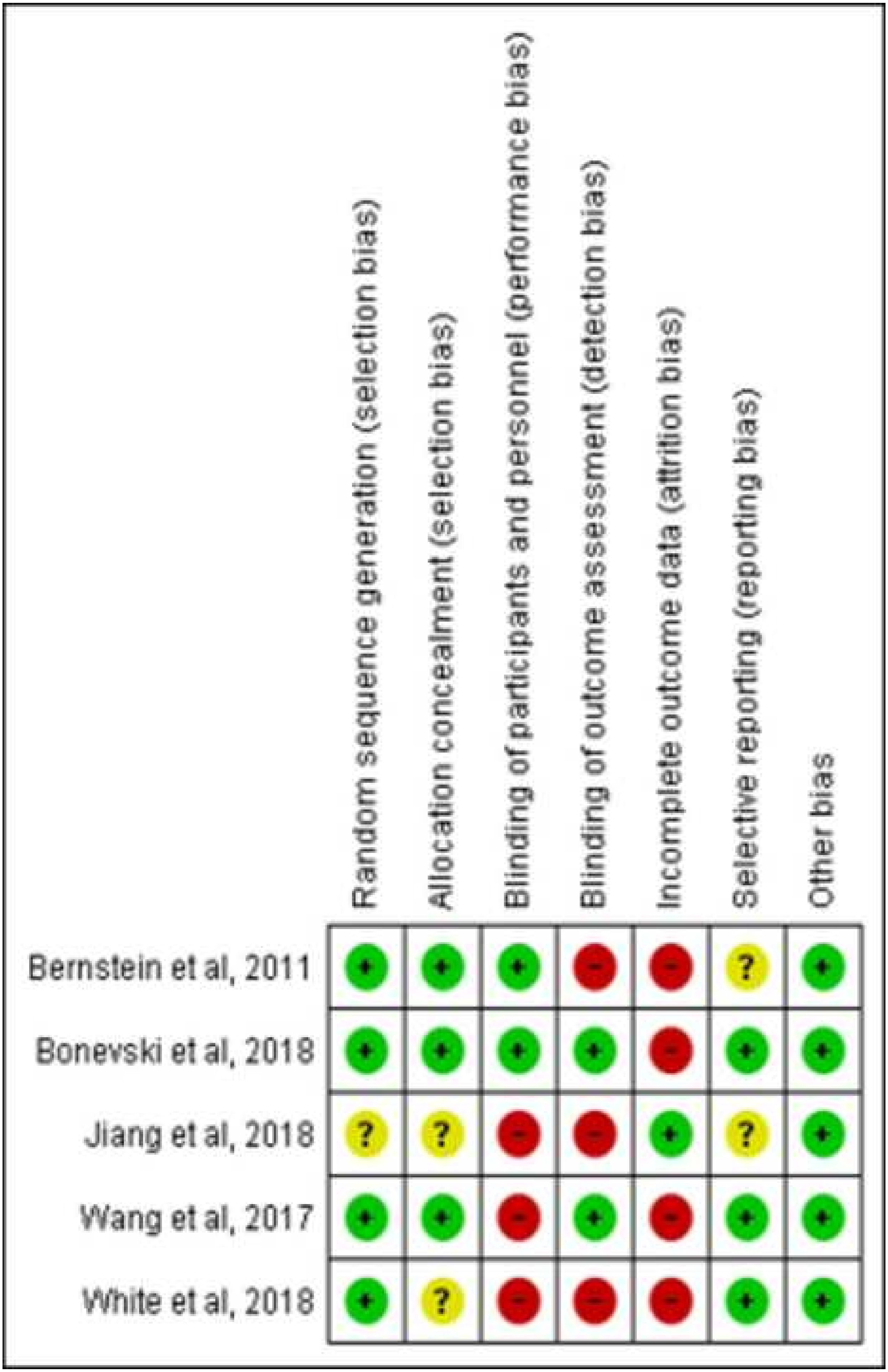

Click here to access/download;Figure;Figure-3b.tif

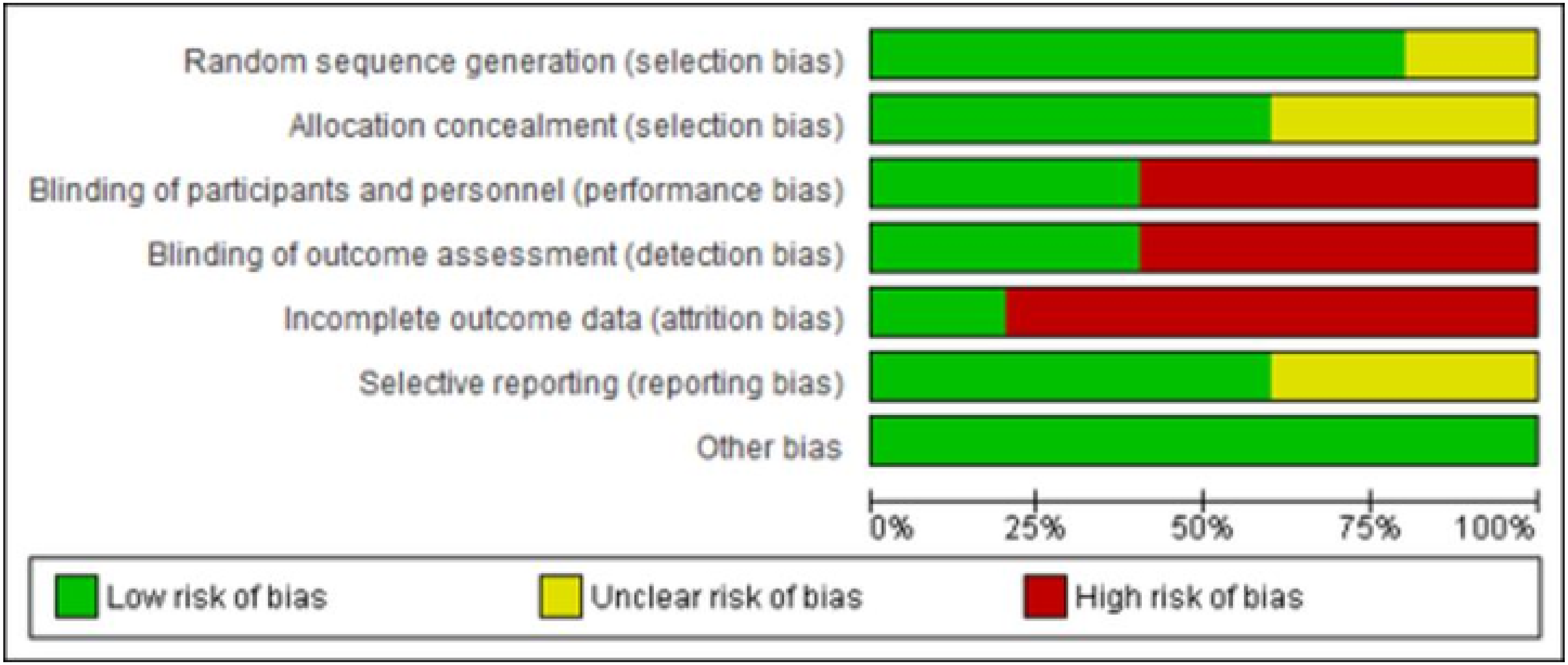

Click here to access/download
Supporting Information
S1 PRISMA Checklist.doc

Click here to access/download
Supporting Information
S2 Risk of Bias Assessment.docx

Effectiveness of Community Health Workers Involvement in Smoking Cessation Programme:; A Systematic Review.

Siti Hafizah Zulkiply[1]*, Lina Farhana Ramli[1], Zul Aizat Mohamad Fisal[1], Bushra Tabassum[1], Rosliza Binti Abdul Manaf*.

[1] Master of Public Health Candidate, Faculty of Medicine and Health Sciences, University Putra Malaysia

*Corresponding authors: Siti Hafizah Binti Zulkiply, hafizahzulkiply.hz@gmail.com

Rosliza Binti Abdul Manaf, rosliza_abmanaf@upm.edu.my

Formatted: Font color: Auto

Formatted: Superscript

Formatted: No underline, Font color: Auto

Formatted: No underline, Font color: Auto

Field Code Changed

~~ABSTRACT~~ Abstract

Formatted: Heading 1

Background: Sustainable Development Goals (SDG) has set the target to reduce ~~by one-third~~ premature mortalities from non-communicable diseases (NCD) by one-third. One of the ways to achieve this is through strengthening the countries' implementation of the World Health Organization Framework Convention on Tobacco Control (WHO FCTC). Community health workers (CHWs) involvement has shown~~ed~~ promising results in the prevention of NCDs. This systematic review is aimed at critically evaluating the available evidence~~s~~ on the effectiveness of involving ~~vement of~~ CHWs in smoking cessation.

Materials and Methods: We systemically searched PubMed and CENTRAL up to September 2019. We searched for published interventional studies on smoking cessation interventions using the usual care that complemented with CHWs as compared to the usual or standard care alone. Our primary outcome wa~~i~~s abstinence of smoking. Two reviewers independently extracted data and assessed study risks of bias.

Result: We identified 2794 articles, ~~w~~of which~~ith~~ only five included studies. A total of 3512 smokers with 41 CHWs were included in the studies. The ~~I~~intervention duration range from 6 weeks to 30 months. The studies used behavioral intervention or a combination of behavioral intervention and pharmacological treatment. Overall, the smoking cessation intervention that incorporate~~s~~d involvement of CHWS ha~~s~~d higher smoking cessation rates [OR 1.95, 95% CI (1.35,2.82)]. Significant smoking cessation rates were seen in two studies.

Conclusion: Higher smoking cessation rates we~~a~~re seen in the interventions that combined the usual care with interventions by CHWs as compared to the usual care alone. However, there were insufficient studies to prove the effectiveness. In addition, there was high heterogeneity in terms of interventions and participants in the current studies. ~~Therefore,~~

CHWs have the potential to strengthen WHO FCTC and reduce the NCD mortalities attributed by smoking which fulfills the SDG target 3.4 and 3a.

Keywords: Community Health Worker, Smoking Cessation, Sustainable Development Goals, Primary Care

Introduction

Non-communicable diseases (NCD) areis the major cause of mortalities worldwide. Smoking is an important risk factor for the development of NCD, including cancers, and cardiovascular and respiratory diseases (1)(). The United Nation General Assembly has developed an aAgenda for the Sustainable Developmental Goals (SDG) in 2015, that containings a total of 17 Goals that all Member States have agreed to achieve by 2030 (2). SDG 3, with goal the to "ensure healthy lives and promoteing well-being for all ages"", includes target 3.4, which is to reduce by one-third premature mortality from NCD by one-third, and target 3a, which is to strengthen a country's implementation of the World Health Organization Framework Convention on Tobacco Control (WHO FCTC) (2,3).

In September 2005, Malaysia had participateded with WHO FCTC and agreed to fulfil the demand to develop and disseminate a standard national guideline on tobacco (4). In response, government of Malaysia (GoM) n government had set up a target to reduce 30% relative reduction of national smoking prevalence by 2025 within the National Strategic Plan for NCD (NSP-NCD). In addition, the World Health Organization (WHO) has also developed MPOWER that include measures for demand reduction: which were; i) Monitoring tobacco use and prevention policies;, ii) Protecting people from tobacco smoke;, iii) Offering help to quit tobacco use;, iv) Warning about the dangers of tobacco,; v) Enforcinge bans on tobacco advertising, promotion and sponsorship; and vi) Raisinge taxes on tobacco (5).

Tobacco has become a major public health enemy, accountable for more than 7 million preventable deaths yearly, and it has been forecasted that more than 8 million people will die from diseases related to tobacco use by year 2030 if pattern of smoking continues (6). In Malaysia alone, it is estimated that more than 20,000 have died due to tobacco each year (7). There are 933.1 million smokers worldwide in 2015 and around 80% of them live in lLow and

Formatted: Font color: Auto

mMiddle-income cCountries (LMICs) (World Health Organization Media Center, 2017). According to the National Health and Morbidity Survey (NHMS) 2015, the prevalence of smokers in Malaysia wasere 22.8%, therefore, it is estimated that nearly five million Malaysians aged 15 years and above are smokers (4). The mean age of smoking initiation iswas 18.3 years (9), with the highest prevalence of smokers beingwere among the 25 to 44- years--old age group (28%), followed by the 45 to 64--years--old age group (20%) (4).

In many countries, smoking cessation programsme (identification of smokers, advisinged and offering ed quit support to quit) are taking place in primary care settings (10). Evidence for the effectiveness of these interventions in this setting administeredgiven by professional healthcare providers is well established (11). Since 2004, Malaysia has set up quit--smoking clinics since 2004 that is available at almost allmost primary health clinics (12). Unfortunately, studies have also shown that high mortalities in low socioeconomic areas, especially secondary to NCD are due to barriers in healthcare services (13). In view ofSince the -low socioeconomic status being is an important determinant of smoking (14), therefore, it is pertinent that interventions on smoking should focus on these populationsshould be given on interventions in smoking cessation in these area. As reported by Global Adult Tobacco Survey (NHMS GATS) 20115, the prevalence of smokers in rural areas were was higher (24.37.7%, 95% CI 22.0, 26.7) as compared to urban area (22.70.9%, 95% CI 20.2, 25.4) (15). The WHO has developed an effective community-based strategy to reduce the gap in healthcare in low-socioeconomic areas. FurthermoreHowever, the, current smoking cessation services were are not felt to be user-friendly and were poorly understood (16).

In line with the Alma Ata declaration of the Primary Healthcare Concept, all community health strategy designs must address the community needs at the local level, and be led by the

Field Code Changed

Field Code Changed

Formatted: Font color: Auto

Field Code Changed

Formatted: Font color: Auto

community members themselves, and require involvement of communities to mobilize local resources. Task shifting, an idea conceived by the WHO, is defined as redistributing primary care responsibilities from physician to non-physician providers (17). Task shifting can be further extended to include health workers without formal healthcare training, known as community health workers (CHWs) (18). In 1989, a WHO study group had established a widely accepted definition of CHWs: the workers ~~that mentioned CHWs~~ should be among the communities where they practice, ~~should~~ be chosen by the communities, be ~~should be~~ able to answer to the public for their programs, be ~~should be~~ supported by the health system, and have less duration of training than professional workers (19). CHWs have the potential to achieve primary health care goals,~~,~~ by enhancing access to care and promote a proper use of health resources through the provision of cultural and outreach links between communities and health systems, giving health education to reduce cost; and,~~,~~ screening services, basic emergency services, continuum of care and client protection (19). Furthermore, it is ~~pertinent~~ imperative to enhance civic engagement and ensure accountability in order to achieve the SDG goals (20,21). There are considerable evidences supporting positive impacts of CHWs on the health of diverse populations especially on the maternal and child health, malaria and tuberculosis. Numerous studies have also evaluated the role of community intervention on major cardiovascular events and risk factors. Community-~~–~~based cardiovascular health intervention i~~i~~n vulnerable populations ~~showed~~ has shown that the interventions aimed at decreasing blood pressure a~~we~~re the most promising and behavior change interventions a~~we~~re the most challenging (22,23). Task-shifting interventions were also proven to be effective in lowering the low-density lipoprotein cholesterol (LDL) and total cholesterol (24). In another review, significant reduction in the mean blood pressure and glycated haemoglobin levels were seen in task-shifting interventions for the CVD risk reduction in LMICs (24,25).

Guidelines on smoking cessation programs~~me~~ ~~showed~~ have shown that the combinations of behavioral change and pharmacological support given by the professional healthcare providers ~~–~~are the most effective. CHWs are~~is~~ crucial as they serve as a critical link in increasing the communities' access to services, especially for people living in rural and undeserved areas, ~~and~~ which ultimately form an integral part needed to achieve the SDG goals. Therefore, the aim of this study is to evaluate the effectiveness of ~~involvement~~ involving ~~of~~ CHWs in smoking cessation programs as compared to the usual care.

Materials and Methods

This review was conducted and reported in accordance to the Preferred Reporting Items for Systematic Reviews and Meta-Analyses (PRISMA) guidelines and based on the Cochrane Collaboration approach ~~()~~(26,27).

Formatted: Font color: Auto

Formatted: Font color: Auto

2.1 Eligibility C~~c~~riteria

We included intervention ~~ntion~~ studies or controlled clinical trials that compared between a combination of interventions given by CHWs and usual care, and ~~with~~ only usual care. ~~We included studies~~Only interventional studies ~~that were~~ conducted among smokers aged 18 years old and above, having the ~~. We included studies with abstinence of smoking~~smoking abstinence rate as the outcome were included. We excluded studies that conducted intervention in adolescent or special group (defines as mental illness or LGBTQ). Non-English language studies, reviews, proceedings, qualitative studies, descriptive studies and protocol were also excluded.

Formatted: Font color: Auto

Formatted: Font color: Red

2.2 Data S~~S~~ources and S~~s~~earch S~~s~~trategy

We systematically searched for relevant articles published ~~-~~in The Cochrane Central Register of Controlled Trials (CENTRAL) and PubMed/MEDLINE up to September 2019. The s~~S~~earch was~~ere~~ limited to 10 years, from 2009 until 2019. We combined the keywords for smoking, cessation and community as the following: cessation or reduction AND smoking or tobacco AND volunteer or peer or community or lay.

2.3 Study S~~s~~election

A pair of authors independently assessed the titles and abstracts of a defined set of articles. Each study was recorded as include, exclude or unclear. The ~~F~~full articles were retrieved for further assessment if they were recorded as include or unclear. Eligible studies were identified based on the inclusion criteria. Any discrepancies in the assessment were resolved by discussion leading to a consensus.

2.4 Data Extraction, Data Analysis and Risk of Bias A~~a~~ssessment

~~A table for data extraction from all potential studies were built~~Data extraction from all potential studies was documented in a table. The table included information on study characteristics (sample size and study duration), participant characteristics (setting, ~~and~~ population type, and specific ethnicity), intervention characteristics (type of interventions given in both arms, and behavioural or pharmacological intervention), training of CHWs (background, duration of training and training module) and analysis and results (outcomes) of both arms. All authors independently extracted the data and any discrepancies were resolved by discussion. The characteristics of the included studies are outlined in Table 1~~, Table 2, Table 3 and Table 4~~ through Table 3.

Formatted: Font color: Auto

Data synthesis and analysis were carried out using Review Manager Software (Rev Man) version 5.3 (Nordic Cochrane Centre, Cochrane Collaboration, Copenhagen). The e~~E~~nd of ~~intervention~~ study values were taken. If studies measured smoking cessation rates at multiple intervals (3 or 6 or 9 months), the ~~-~~outcomes i~~o~~n the final point of interval were~~as~~ taken. If studies reported two types of smoking cessation measurements (self-reported or ~~validated~~chemically verified using carbon monoxide (CO) level), the chemically verified~~validated~~ outcomes were taken.

We assessed the study quality of each ~~included~~ study using the COCHRANE guideline for assessment of systematic reviews and the published assessment guide on risks of bias assessment for intervention studies (26). The studies were evaluated based on eight criteria: randomized treatment order, allocation concealment, blinding of participants and personnel, blinding of outcome assessment, incomplete outcome data, selective outcome reporting and other bias. For each item, risk of bias was classified as 'low risk', 'high risk, or 'unclear risk', with the last category indicating either a lack of information or uncertainty over the potential for bias. The r~~R~~esults were presented in a 'Risk of bias' summary (Figure 3a and 3b) and S2 Table.

Result

3.1 Search R~~r~~esults

We identified 2794 articles through our electronic databases~~s~~ search. After excluding 280 duplicated studies, ~~A~~a total of 261~~9~~4 articles were excluded following titles and abstracts screening due to irrelevant study designs (observational studies or interventional studies without control group), irrelevant ~~irrelevant study populations,~~ outcomes (no smoking cessation or did not provide smoking abstinence rate), and irrelevant interventions (not given by CHWs)~~intervention characteristics~~. One hundred~~100~~ full text articles were screened, of

Formatted: Font color: Auto

Formatted: Font color: Auto

Formatted: Font: Not Italic

which 95 articles were excluded for following reasons: ~~;~~ interventions were not given by CHWs (73 studies), study designs were non~~-randomized~~ controlled trials (17 studies) and outcomes were not smoking cessation (5 studies). Finally, five studies we~~a~~re included in this review (28–32).~~-~~ Figure 1 shows PRISMA f~~F~~lowchart~~ can be found at Figure 1~~.

Formatted: Font color: Auto

Figure 1: PRISMA flowchart for the selection of studies. Outcomes of the systematic review of the literature by record identification, screening, and analysis in the Preferred Reporting Items for Systematic Reviews and Meta-Analyses (PRISMA) statement flow diagram.

Formatted: Font: 12 pt

3.2 Characteristics of I~~i~~ncluded S~~s~~tudies

A total of 3512 participants we~~a~~re included in this review. The S~~s~~tudies were conducted between the year of 2011 and 2018. Two studies were conducted in United States of America (USA) (28,29)~~,~~ while the other studies were conducted in Hong Kong (30), Australia (32) and Vietnam (31)~~ respectively~~. ~~D~~The duration of the studies conducted ~~were~~ ranged~~ing~~ from 6 weeks to 30 months. All studies were conducted among the community, except for one study ~~that~~ conducted in an emergency department setting (28). The same~~One~~ study ~~were targeted~~was conducted on a specific ethnicities~~y~~ (African American and Hispanic community) (28). There were no significant differences between the baseline number of cigarettes per day (13.08 ± 9.57).

Table 1. Characteristics of the studies

Study	Setting	Participants type	Sample size	Intervention	Control	Mean cigarettes/ day	Study Design	Study Duration
**Bernstein *et al*, 2011** (28)	Bronx, New York, United States of America	Hispanic and African American	337	170	167	15 ± 7.48	RCT	21 months
**Wang *et al*, 2017** (30)	Hong Kong	Public	1,226	402	416<br>408	N/A	Cluster-3 arms RCT	3 months
**White *et al*, 2018** (29)	Maryland, United States of America	Public	200	101	99	19.3 ± 18.3	RCT	6 – 8 weeks
**Bonevski *et al*, 2018** (32)	New South Wales, Australia	Disadvantage Adult clients of the Community Care Centre	431	187	244	15 ± 7.46	Parallel randomised trial	30 months
**Jiang *et al*, 2018** (31)	Thai Nguyen, Vietnam	Village Population	1,318	781	537	11.02 ± 9.48	RCT and Quasi-experimental	6 months

Abbreviations: RCT- randomized controlled trial

3.3 Characteristic of Intervention

All of the studies used behavioral intervention such as brief advice ~~-~~as the method for smoking cessation. The usual care intervention ~~that were~~ given to both intervention groups and control group we~~a~~re mainly brief advice, and some of the studies provided self-help material. Four studies used brief advice; one study used AWARD model (Ask, Warn, Advise, Refer and Do-it-again) (30), one study used 4A's model (Ask~~ screen~~ for tobacco use, Advise to quit, Assess readiness to quit, and Assist) (31), one study used automated Smokefree TXT (29) and~~ finally~~ one study used on~~-~~screen advice (32). Self-help materials in the form of leaflet or brochure were~~as~~ provided to the participants in three studies (28,30,31).

The interventions given by CHWs in~~by~~ the studies were behavioral intervention, active referral and pharmacological therapy. Two studies used motivational interviewing (MI), and provided pharmacological treatment (nicotine replacement therapy (NRT)) to the participants (28,32). One study had active referral to the existing smoking cessation intervention in clinics by CHWs (30). One study used personalized text messages (29) as a mediator for smoking cessation intervention. Table 2 shows the d~~D~~etails of the ~~study~~ interventions~~ can be found in Table 2~~.

3.4 Smoking Abstinence Rate

All studies used intention to treat analysis, except for the study by Bonevski et al. (32). The o~~O~~utcomes of the smoking abstinence rate were measured by either self-reporting~~ed~~ or chemically verified ~~(CO~~using CO level~~)~~. Two studies provided abstinence rate a~~t~~fter 3 months (28,29) and the remaining three studies after~~t~~ 6 months (30–32).

The pooled OR were estimated using random effects models as the studies included were heterogenous with respect to interventions and populations. Overall, all studies had higher odds of abstinence in the intervention group as compared to the usual group [OR 1.95, 95% CI (1.35,2.82)]. Two studies reported significant smoking abstinence rate in the interventions using CHWs, with OR 2.96 [2.15, 4.07] (p<0.001) (31), ~~and O~~OR 1.81 [1.04, 3.16] (active referral vs brief advice group), and~~,~~ OR 1.85 [1.06, 3.23] (active referral vs control group) (30). Figure 2 and Table 2 show the ~~D~~details of the result~~ can be found in Table 2~~.

Figure 2: Forest plot shows smoking abstinence rate.

Table 2. Characteristic of Intervention and Outcome

Study	Intervention	Control	Outcome Intervention	Outcome Control
**Bernstein *et al*, 2011** (28)	Smoking cessation brochure (10-15 min) AND MI by interventionist, 6 weeks course of NRT.	Smoking cessation brochure and contact information to smoking cessation programs	Self-reported 7-days PPA rates at 3 months: 14.7%<br>OR 1.14 [0.61, 2.11]	Self-reported 7-days PPA rates at three months: 13.2%
**Wang *et al*, 2017** (30)	Brief advice using structural model AWARD, health warning leaflet AND active referral to SC services by ambassadors	Brief advice using structural model AWARD, health-warning leaflet and encourage to SC services	Validated abstinence rates at 6 months: 9.0% ($p<0.05$)<br>OR 1.81 [1.04, 3.16]<br>OR 1.85 [1.06, 3.23]	Validated abstinence rates at 6 months:<br>5.0% and<br>5.1%
**White *et al*, 2018** (29)	Automated SFTXT<br>AND personalized text messages from peer mentor	Automated SFTXT messages	Self-reported 7-day PPA rates at 3 months: 7.9%<br>OR 2.75 [0.71, 10.70]	Self-reported 7-day PPA rates at 3 months: 3.0%
**Bonevski *et al*, 2018** (32)	On screen advice to quit smoking, state Quitline telephone number, and a gift bag with call it quits<br>AND MI and NRT by trainer volunteer case workers	On screen advice to quit smoking, state Quitline telephone number, and a gift bag with call it quits	Self-reported continuous verified PPA at 6 months: 1.0%<br>OR 0.76 [0.07, 8.44]	Self-reported continuous verified PPA at 6 months: 1.4%
**Jiang *et al*, 2018** (31)	4As (brief counselling and educational materials) AND refer smokers to a trained VHW (4As+R)	4As (brief counselling and educational materials)	Validated abstinence rate at 6 months: 25.7% ($p<0.001$)<br>OR 2.96 [2.15, 4.07]	Validated abstinence rate at 6 months: 10.5%

Abbreviations: MI- Motivational Interviewing; NRT- nicotine replacement therapy; PPA- point prevalence abstinence; SC- smoking cessation; CHW-Community Health Worker; AWARD; SFTXT- SmokefreeTXT; SC- smoking cessation; 4As- Ask (screen for tobacco use), Advise to quit, Assess readiness to quit, and Assist; VHW- Village Health Worker.

3.5 Characteristics and Training of Community Health Workers (CHWs)

The number of CHWs in the study varied from one person to 32 persons;~~,~~ ~~with~~ two studies did not provide the amount of CHWs (30,32). Only one~~Two~~ study~~ies~~ had CHWs from the same ~~communiti~~community with the participants~~es~~ (31).~~,~~ ~~one~~Two studies~~y~~ had ~~university students~~volunteers from NGOs (30,32) ~~and~~ or university students ~~one study had volunteers from NGO~~ (30). CHWs from two studies had prior experiences~~d~~ working as CHWs or worked~~ing~~ on smoking cessation programs previously (29,31). Two ~~One~~ studies~~y~~ had CHWs ~~whom was~~who were former smokers (28,29). ~~and provided incentives to the CHWs (29).~~

The duration of training of the CHWs ~~ranging~~ ranged from just two hours to two weeks. The ~~T~~training ~~were~~ were given by either the researchers or experts in the field. The c~~C~~ontent of the training ~~were~~ was comprised of the study protocol and method in smoking cessation intervention. Supervisions were given weekly or biweekly to ensure fidelity. Only one study provided incentives to the CHWs (29). Table 3 shows the ~~D~~details of the characteristics of CHWs and their training ~~can be found in Table 3~~.

Table 3: Characteristics of CHWs and training of CHWs.

Table 3. Characteristic of Community Health Worker and Training of Community Health Worker

Study	Amount and Description	Educational Background	Experience	Incentive	Duration/ Training Module/ Supervision
**Bernstein *et al*, 2011** (28)	One (Interventionist~~Peer educator~~) Former smoker ED based	Not reported ~~ED based and former smoker.~~	Not reported	None	Duration: 2 weeks<br><br>Training module: Course and practicum (roleplay, slide shows and observation)- Epidemiology, health effects and treatment of tobacco dependence, motivational interviewing.<br><br>Supervision: Every two weeks
**Wang *et al*, 2017** (30)	Not mentioned (SC ambassador)	University students (health-related studies).~~ and volunteers from NGO.~~	Volunteers from NGO~~Not reported~~	None	Duration: 4 hours<br><br>Training module: Tobacco control and SC, SC reduction advice skills<br><br>Supervision: Spot checks
**White et al, 2018** (29)	Thirty-two (Peer mentor) Former smoker more than 1 year 18 years old and above Lived in the United States Willingness to mentor smokers through texting and completing the online training program	~~Former smoker more than 1 year, 18 years old and above and lived in the United States~~,Not reported ~~willingness to mentor smokers through texting and completing the online training program.~~	Facilitators of the American Cancer Society Freshstart group-based cessation support program.	$200 and entry into $1000 drawing	Duration: 2 hours<br><br>Training module: Online- study details, smoking and SC, MI, web-based-text-messaging platform<br><br>Supervision: N/A
**Bonevski et al, 2018** (32)	Not mentioned (Caseworker)	Not reported~~Volunteer case workers of~~	Volunteer case workers of community social	None	Duration: One day<br><br>Training module: Behavioral counselling and MI

Formatted Table

Study	Amount and Description	Educational Background	Experience	Incentive	Duration/ Training Module/ Supervision
		~~community social service organizations (NGO) in NSW.~~	service organizations (NGO) in NSW. ~~Not reported~~		Supervision: N/A
**Jiang et al, 2018** (31)	Six to eight (VHW) Not a current smoker and willing to participate in the required components of the study intervention.	Not reported~~Not a current smoker and willing to participate in the required components of the study intervention.~~	Worked as a VHW at that particular site for a year or more.	None	Duration: 4 days<br><br>Training module: Research ethics, study protocol, use of CO monitor, theory of behavior change, MI, social cognitive skills building approach.<br><br>Supervision: N/A

Abbreviations: N/A- not available; SC-Smoking Cessation; MI-motivational Interviewing; ED- Emergency Department; NGO-non-governmental organizations; NSW-New South Wales; VHW-village health worker.

Formatted Table

3.6 Risk of B~~b~~ias A~~a~~ssessment

We judged the risk of possible bias present in the ~~included~~ studies according to the four incorporated criteria ~~incorporated~~. We presented the summary of risk of bias assessment of the ~~included~~ studies in ~~Table 4~~S2 Table and Figure 3(a) to ~~and~~ 3(b). The r~~Random~~andom sequence generation was assessed to be of low risk of bias in all studies except for the study by Jiang et al. (31) as they did not clearly state the method of random sequence generation. When assessing the allocation concealment of the included studies, three studies (28,30,32) had low risk of bias while the other two studies (29,31) did not clearly state the method of allocatingon ~~of~~ the treatment groups. Performance bias was assessed to be low risk ~~of bias~~ in only two studies ~~study~~ (28,32) with the remaining studies assessed to be high risk ~~of bias~~. The d~~D~~etection bias was assessed to be low risk ~~of bias~~ in two studies (30,32). Only the study by Jiang et al.~~,2018~~ had low risk in attrition bias (31) while~~,~~ the remaining studies were assessed to be high risk ~~of bias~~ due to the high dropout rate (>20%). Three studies with available protocol and all the pre-specified outcomes measured were considered as low risk of reporting bias (29,30,32).

Formatted: Font color: Auto

Field Code Changed

Field Code Changed

Field Code Changed

Field Code Changed

Field Code Changed

Formatted: Font color: Red

Figure 3 (a) and 3 (b): Risk of bias assessment.

Discussion

This review evaluated the effectiveness of involving CHWs in smoking cessation programs as compared to the usual care. Our review found higher smoking cessation rates in the interventions involving CHWs as compared to usual care in five studies. There are considerable evidences supporting impact of CHWs on the health of diverse populations especially on maternal and child health, malaria and tuberculosis. However, eEvidence of effectiveness of CHWs in the cardiovascular diseases (CVD) management and prevention isare still lacking. Previous studies reported evidences on the four main risk of CVDs:; namely hypertension, diabetes mellitus, unhealthy diet and alcohol and tobacco consumption. Task sharing intervention were proven effective in lowering low-density lipoprotein cholesterol (LDL) and total cholesterol (31). In another review, significant reduction in mean blood pressure and glycated haemoglobin levels were seen in task shifting interventions for CVD risk reduction in LMICs (31,32). A study by Jeet et al., 2017 also reported an increase in tobacco cessation in interventions using CHWs as compared with the standard care (33), as with this review. Our review has also found similar finding, promising evidence in interventions given by CHWs where higher smoking cessation rates were seen.

The objective of having CHWs is having the intervention given by someone from the community itself. Its effectiveness vary depending on their training program, demographics and settings (34). Significant smoking cessation rates were seen in two studies that conducted the training in 4 hours (30) and 4 days (31). Only one study had CHWs from the same community with the participants, and reported significant reduction of smoking cessation rate. In addition, the study only takes CHWs that have prior experience working as CHWs at that particular site. The performance of CHWs is also associated with their age, skill and educational level (35). Another study that reported significantly higher cessation rate had university students or volunteers from NGOs as the CHWs (30).

The WHO has formulated a set of recommendations that provides guidance for the task-shifting approaches. These recommendations have implications for a range of health services including the –management of NCDs. The mMain enablers of CHWs interventions are provision of algorithms and protocols, while, restrictions on prescribing medications and availability of medicines are thewere main barriers identified. Therefore, to ensure its effectiveness, a sound protocol should be developed. However, there was a high heterogeneity in the interventions given among the included studies. The guidelines on smoking cessation programs showed that a combination of behavioral change and pharmacological support are the most effective. All of the studies used behavioural intervention as the method for smoking cessation with two studies providing pharmacological treatment (nicotine replacement therapy) to the participants. Our review showed that significant higher abstinence rates were seen in one study that used active referral, AWARD and leaflet (30) and one study that used 4A's (31) as the method for behavioural intervention.

CHWs productivity is also largely determined by the condition under which they work. The focus on the provision of an enabling work environment for CHWs is essential for achieving high levels of productivity. Jaskiewicz et al. presented a model in which the work environment encompassed four essential elements: workload, supportive supervision, supplies and equipment, and respect from the community and the health system (36). Similarly, important determinant of positive CHWs interventions is community embeddedness, meaning that community members have a sense of ownership of the program (37).

There are several limitations in this review.~~,~~ F~~f~~irst is the high heterogeneity in baseline characteristics of the participants and study designs. Theoretical health belief models a~~explained~~ ssert that a person who is in contemplation stage may be easily influenced to receive intervention. Therefore, the studies with the participants ~~with~~ of low exposure to smoking and of intention to quit ~~participants~~ (theoretical health belief models) might be attributed~~able~~ to higher cessation rates. One study that selected participants who already had~~ve~~ the intention to quit smoking showed similar results (29). Similarly, one study that included participants with low ~~-~~amount of cigarettes used (one in the past 3 months or 1 months) showed significant smoking cessation rates (30). ~~W~~Meanwhile, one study ~~that was~~ conducted in a hospital setting (emergency department) reported higher abstinence rate in smokers who had~~ve~~ smoking-related health issues (28). ~~R~~The review of 33 studies reported high-certainty evidence that incentives improve smoking cessation rates (38). Our review showed similar result, whereby one study that provided incentives to the participants had significant smoking cessation rates (30).

~~There was also high heterogeneity in the interventions given. Guidelines on smoking cessation programme showed that combination of behavioral change and pharmacological support are the most effective. All of the studies used behavioural intervention as the method for smoking cessation with two studies provided pharmacological treatment (nicotine replacement therapy) to the participants. Our review showed that significant higher abstinence rates were seen in one study that used active referral, AWARD and leaflet (24) and one study that used 4A's (25).~~

~~The effectiveness of using CHWs vary depending on their training program, demographics and settings (31). CHWs productivity is determined in large part by the condition under which~~

~~they work. Attention to the provision of an enabling work environment for CHWs is essential for achieving high levels of productivity. Jaskiewicz et al. present a model in which the work environment encompasses four essential elements, namely workload, supportive supervision, supplies and equipment, and respect from the community and the health system (32). Another important determinant of positive CHWs interventions is community embeddedness whereby community members have a sense of ownership of the program (33). Significant smoking cessation rates were seen in two studies that conducted the training in 4 hours (24) and 4 days (25) respectively. However, other factors have to be considered. Performance of CHWs were also associated with their age, skill and educational level (34). Study that reported significant higher cessation rate had university students as the CHWs (Wang et al., 2017). While, another study that reported significant higher cessation rate had CHWs from the community and volunteer from NGOs(25). The CHWs from this study had experienced working as CHWs previously (25) .~~

Higher retention i~~wa~~s significantly associated with higher quit rates. However, there were high attrition rates (loss of follow up of >20%) in most of the studies included in this review, with the highest attrition rates of 42% (32). The r~~R~~eviews reported that increasing age, higher level of education and higher motivation to quit were associated with higher retention (39).

Recommendations

Previous review has reported cost-effectiveness of CHWs interventions as compared to standard care particularly in tuberculosis, malaria and maternal and child health (40,41). We recommend ~~that~~ future studies to ~~analyze~~analyse the cost-effectiveness of using CHWs in smoking cessation programs. ~~In view of~~Since the effectiveness of CHWs intervention is largely dependent on the framework and training module, ~~therefore, it is pivotal~~it is

Formatted: Space After: 0 pt

imperative ~~-~~that the guidelines on the framework are~~is~~ drawn up. We also recommend~~ that~~ studies to measure~~s~~ abstinence rates of smoking according to the gold standard.

Conclusion

CHWs have the potential to ~~be a~~ bridge between primary healthcare providers and communities, and ~~therefore~~ consequently reducing~~e~~ the gap, especially among low socioeconomic populations as recommended by the SDG. Our review reported~~–~~ positive outcomes on using a ~~-~~combination of CHWs and usual care in smoking cessation as compared with usual care alone, however the evidences are insufficient and have high heterogeneity.

Acknowledgement

We would like to thank the Director General of Health Malaysia, Director of Institute for Medical Research for the permission to publish this article. ~~We would also would like to thank Prof. Madya Dr. Rosliza Abdul Manaf for the guidance and support.~~

Declaration

The authors declare no conflict of interest.

Funding

The authors received no specific funding.

~~Supplementary Information~~

~~S1 PRISMA Checklist~~

~~S2 Risk of Bias Details~~

Authors C~~c~~ontribution

Siti Hafizah Zulkiply: Analysis, Writing- Original draft and final editing.

Lina Farhana Ramli: Analysis- Writing - Original draft

Zul Aizat Mohamad Fisal:~~ Analysis, ~~ Writing- Original draft

Bushra Tabassum: Analysis, Writing- Original d~~D~~raft

Rosliza Abdul Manaf: Concept development and final editing.

References

1. Glantz S, Gonzalez M. Effective tobacco control is key to rapid progress in reduction of non-communicable diseases. The Lancet. 2012.
2. United Nation. Transforming Our World: The 2030 Agenda for Sustainable Development. In: A New Era in Global Health. 2015.
3. World Health Organisation. Who Framework Convention. WHO Framew Conv Tob Control [Internet]. 2003;42. Available from: http://www.who.int/tobacco/framework/WHO_FCTC_english.pdf
4. Institute for Public Health. National Health and Morbidity Survey 2015 (NHMS 2015). Ministry of Health Malaysia. 2015.
5. World Health Organization. MPOWER : Six policies to reverse the tobacco epidemic. WHO Rep Glob Tob Epidemic. 2008. p ;23–41.
6. WHO. Monitoring tobacco use and prevention policies fresh and alive. 2017. p 1–135 p.
7. Ministry of Health Malaysia. Clinical Practice Guidelines on Treatment of Tobacco Use Disorder. 2016.
8. Worllrd Health Organization Media Center. WHO Fact Sheet | Tobacco. Wolrld Health organization Fact Sheet. 2017.
9. Lim HK, Ghazali SM, Kee CC, Lim KK, Chan YY, Teh HC, et al. Epidemiology of smoking among Malaysian adult males: Prevalence and associated factors. BMC Public Health. 2013;13(1):2–11.
10. Piné-Abata H, Mcneill A, Murray R, Bitton A, Rigotti N, Raw M. A survey of tobacco dependence treatment services in 121 countries. Addiction. 2013;108(8):1476–84.
11. Stead LF, Buitrago D, Preciado N, Sanchez G, Hartmann-Boyce J, Lancaster T. Physician advice for smoking cessation. Cochrane Database of Systematic Reviews. 2013.
12. Fai SC, Yen GK, Malik N. Quit rates at 6 months in a pharmacist-led smoking cessation service in Malaysia. Can Pharm J. 2016 Sep;149(5):303–12.
13. Yusuf S. Improving worldwide access to inexpensive and effective treatments for common cardiovascular diseases. Eur Hear Journal, Suppl. 2018;20:C18–22.
14. Hiscock R, Dobbie F, Bauld L. Smoking cessation and socioeconomic status: An update of existing evidence from a national evaluation of english stop smoking services. Biomed Res Int. 2015;2015.
15. Mohd Yusoff MF, Guat Hiong HT, Singh S, Kuang Hock L. Report of the global adult tobacco survey (GATS). Inst Public Heal. 2011;204.
16. Chean KY, Goh LG, Liew KW, Tan CC, Choi XL, Tan KC, et al. Barriers to smoking cessation: A qualitative study from the perspective of primary care in Malaysia. BMJ Open. 2019;9(7):1–8.
17. World Health Organization. Task Shifting- Global Recommendations & Guidelines. World Heal Organ. 2008;
18. Fulton BD, Scheffler RM, Sparkes SP, Auh EY. Human Resources for Health Health workforce skill mix and task shifting in low. Hum Resour Health. 2011;1–11.
19. Lehmann U, Sanders D. Community health workers: What do we know about them? 2007.
20. Buse K, Hawkes S. Health in the sustainable development goals: Ready for a paradigm shift? Global Health. 2015;11(1):1–8.
21. Joshi R, Peiris D. Task-sharing for the prevention and control of non-communicable diseases. Lancet Glob Heal [Internet]. 2019;7(6):e686–7. Available from: http://dx.doi.org/10.1016/S2214-109X(19)30161-5
22. Joshi R, Thrift AG, Smith C, Praveen D, Vedanthan R, Gyamfi J, et al. Task-shifting for cardiovascular risk factor management: Lessons from the Global Alliance for Chronic Diseases. BMJ Glob Heal. 2018;3:1–8.
23. Anand TN, Joseph LM, Geetha A V., Prabhakaran D, Jeemon P. Task sharing with non-physician health-care workers for management of blood pressure in low-income and middle-income countries: a systematic review and meta-analysis. Lancet Glob Heal [Internet]. 2019;7(6):e761–71. Available from: http://dx.doi.org/10.1016/S2214-109X(19)30077-4

24. Anand TN, Joseph LM, Geetha A V., Chowdhury J, Prabhakaran D, Jeemon P. Task-sharing interventions for cardiovascular risk reduction and lipid outcomes in low- and middle-income countries: A systematic review and meta-analysis. J Clin Lipidol. 2018;12(3):626–42.
25. Ogedegbe G, Gyamfi J, Plange-Rhule J, Surkis A, Rosenthal DM, Airhihenbuwa C, et al. Task shifting interventions for cardiovascular risk reduction in low-income and middle-income countries: A systematic review of randomised controlled trials. BMJ Open. 2014;4(10).
26. Higgins JPT GS (editors). Cochrane Handbook for Systematic Reviews of Interventions Version 5.1.0 . The Cochrane Collaboration . 2011.
27. Liberati A, Altman DG, Tetzlaff J, Mulrow C, Gøtzsche PC, Ioannidis JPA, et al. The PRISMA statement for reporting systematic reviews and meta-analyses of studies that evaluate health care interventions: explanation and elaboration. In: Journal of clinical epidemiology. 2009.
28. Bernstein SL, Bijur P, Cooperman N, Jearld S, Arnsten JH, Moadel A, et al. A randomized trial of a multicomponent cessation strategy for emergency department smokers. Acad Emerg Med. 2011;
29. White JS, Toussaert S, Thrul J, Bontemps-Jones J, Abroms L, Westmaas JL. Peer Mentoring and Automated Text Messages for Smoking Cessation: A Randomized Pilot Trial. Nicotine Tob Res. 2019;1–10.
30. Wang MP, Suen YN, Li WHC, Lam COB, Wu SY Da, Kwong ACS, et al. Intervention with brief cessation advice plus active referral for proactively recruited community smokers: A pragmatic cluster randomized clinical trial. JAMA Intern Med. 2017;177(12):1790–7.
31. Jiang N, Siman N, Cleland CM, Van Devanter N, Nguyen T, Nguyen N, et al. Effectiveness of Village Health Worker–Delivered Smoking Cessation Counseling in Vietnam. Nicotine Tob Res. 2018;1–7.
32. Bonevski B, Twyman L, Paul C, D'Este C, West R, Siahpush M, et al. Smoking cessation intervention delivered by social service organisations for a diverse population of Australian disadvantaged smokers: A pragmatic randomised controlled trial. Prev Med (Baltim) [Internet]. 2018;112(November 2017):38–44. Available from: https://doi.org/10.1016/j.ypmed.2018.04.005
33. Jeet G, Thakur JS, Prinja S, Singh M. Community health workers for noncommunicable diseases prevention and control in developing countries: Evidence and implications. PLoS One [Internet]. 2017;12(7):1–21. Available from: http://dx.doi.org/10.1371/journal.pone.0180640
34. Abdel-All M, Putica B, Praveen D, Abimbola S, Joshi R. Effectiveness of community health worker training programmes for cardiovascular disease management in low-income and middle-income countries: A systematic review. BMJ Open. 2017;7(11):1–11.
35. Chung MHL, Hazmi H, Cheah WL. Role performance of community health volunteers and its associated factors in Kuching district, Sarawak. J Environ Public Health. 2017;2017.
36. Jaskiewicz W, Tulenko K. Increasing community health worker productivity and effectiveness: A review of the influence of the work environment. Hum Resour Health. 2012;10:1–9.
37. Scott K, Beckham SW, Gross M, Pariyo G, Rao KD, Cometto G, et al. What do we know about community-based health worker programs? A systematic review of existing reviews on community health workers. Hum Resour Health. 2018;16(1):1–17.
38. Notley C, Gentry S, Livingstone-Banks J, Bauld L, Perera R, Hartmann-Boyce J. Incentives for smoking cessation. Cochrane Database of Systematic Reviews. 2019.
39. Courtney RJ, Clare P, Boland V, Martire KA, Bonevski B, Hall W, et al. Predictors of retention in a randomised trial of smoking cessation in low-socioeconomic status Australian smokers. Addict Behav [Internet]. 2017;64:13–20. Available from: http://dx.doi.org/10.1016/j.addbeh.2016.07.019
40. Nkonki L, Tugendhaft A, Hofman K. A systematic review of economic evaluations of CHW interventions aimed at improving child health outcomes. Hum Resour Health. 2017;15(1):1–19.
41. Vaughan K, Kok MC, Witter S, Dieleman M. Costs and cost-effectiveness of community health workers: Evidence from a literature review. Hum Resour Health. 2015;13(1).

Supporting Information

S1 PRISMA Checklist

S2 Risk of Bias Details
