# Supplementary material for: Effectiveness of community health workers involvement in smoking cessation programme: A systematic review
Source: PLoS One. 2020 Nov 19;15(11):e0242691. doi: 10.1371/journal.pone.0242691 (PMC7676728; doi:10.1371/journal.pone.0242691)
Supplement: Attachment — Submitted filename: Response to Reviewers.docx [file pone.0242691.s005.docx]

Dear Reviewers,

Thank you for your kind and deliberate comments. Please find below the responses to your comments.

Reviewer #1: The paper still contains editing mistakes, please have a further review. For example:

Line 49 'with goal the to “ensure”’

Line 49 has been edited

Line 57 'to reduce 30% relative reduction’

Line 57 has been edited

Line 267 roleplay

Line 267 has been edited

The following comments are related to the tables, please review your tables of results and adjust all the necessary information:

Bernstein et all has 168 patients in the control group, but the table 1 reported 167, the values reported in the study for cigarettes/day were median and IQR for the sample of 168, but the ones reported in the table are Mean and SD for 167.

The n for the control group has been changed to 168 and we have recalculated the mean and sd accordingly.

Table S2 reports 26% of incomplete outcome data, was this related with the 26+31 missing information and/or the expired information over the total sample of the randomized data (338)? if so, the estimation is not correct.

The percentage of loss to follow up has been recalculated and updated in the Table S2 and Figure 3 (a) and 3(b).

Please verify the results of the OR reported data. In table 2, Bonevski et al, 2018, we observe that the OR from the Self-reported continuous verified PPA at 6 months is 0.76, while in the paper the "continuous self-reported PPA at 6 months” is 1.95 and the OR from the "Continuous verified PPA at 6 months" is 0.77.

The outcome for Bonevski et al has been changed accordingly.

Please review the results and forest plot
Table 2, reference 29, the Biochemically verified abstinence at 3 months is 8/101 in the intervention group and 3/99 in the control group. The self-reported abstinence at 3 months was 24/101 in the intervention and 13/99 in the control group. If you consider to add both, please include the information.

We have made the correction and only included the biochemically verified outcome. The forest plot has been adjusted accordingly in Figure 2.

Figure 2. Taking into account that the Jiang et al study presents 25.7% of abstinences rate, should the number of events in the CHW group be 201 instead of 200?

The number of events for Jiang et al has been corrected.
